# From pancreas and islet resources to diabetes insights

**DOI:** 10.1007/s00125-026-06731-4

**Published:** 2026-05-01

**Authors:** Sing-Young Chen, Christiana Lekka, Jonas Lemvig, Xiaoyan Yi, Miriam Cnop, Piero Marchetti, Lorella Marselli, Jianguo Xia, Jesper G. S. Madsen, Teresa Rodriguez-Calvo, Sarah J. Richardson, James D. Johnson, Patrick E. MacDonald

**Affiliations:** 1 Life Sciences Institute, Department of Cellular and Physiological Sciences, University of British Columbia, Vancouver, BC Canada; 2 Department of Surgery, University of British Columbia, Vancouver, BC Canada; 3 Islet Biology Exeter (IBEx), Exeter Centre of Excellence for Diabetes Research (EXCEED), Department of Clinical and Biomedical Sciences, University of Exeter Medical School, Exeter, UK; 4 Functional Genomics and Metabolism Research Unit, Department of Biochemistry and Molecular Biology, University of Southern Denmark, Odense, Denmark; 5 ULB Center for Diabetes Research, Medical Faculty, Université Libre de Bruxelles, Brussels, Belgium; 6 Division of Endocrinology, Erasmus Hospital, Université Libre de Bruxelles, Brussels, Belgium; 7 WEL Research Institute, Wavre, Belgium; 8 Department of Clinical and Experimental Medicine, University of Pisa, Pisa, Italy; 9 Institute of Parasitology, McGill University, Montreal, QC Canada; 10 Department of Microbiology and Immunology, McGill University, Montreal, QC Canada; 11 German Center of Diabetes Research (DZD), Institute of Diabetes Research, Helmholtz Zentrum München, German Research Center for Environmental Health, Munich-Neuherberg, Germany; 12 Department of Internal Medicine – Metabolism, Endocrinology & Diabetes (MEND), Faculty of Medicine, University of Michigan, Ann Arbor, MI USA; 13 Lund University Diabetes Centre (LUDC), Department of Clinical Sciences – Malmö, Faculty of Medicine, Lund University, Lund, Sweden; 14 Department of Pharmacology, Faculty of Medicine and Dentistry, University of Alberta, Edmonton, AB Canada; 15 Alberta Diabetes Institute, University of Alberta, Edmonton, AB Canada

**Keywords:** Biobanking, Data repositories, Diabetes, Islets of Langerhans, Knowledge bases, Multi-omics, Open science, Review, Standardisation, Tissue phenotyping

## Abstract

Pancreatic islets of Langerhans are central to the pathogenesis of all major forms of diabetes. The ability to study human islets ex vivo has advanced our understanding of diabetes and aided in the development of novel therapeutics. However, for decades, very few laboratories had access to this critical resource and experiments on human islets were typically underpowered. More recently, multiple consortia around the world have started to enable islet biology at scale, enriching our understanding of the intra-individual variability of islet function and disease mechanisms. This article reviews and compares existing large-scale human islet tissue and data resources, offering suggestions for their improvement and for developing new resources.

**Graphical Abstract:**

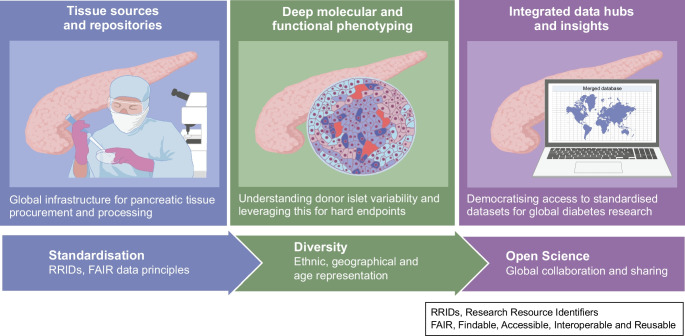

**Supplementary Information:**

The online version contains a slideset of the figures for download available at 10.1007/s00125-026-06731-4.

## Introduction

Early histopathology [1], small-scale tissue isolation [2, 3], advances in semi-automated pancreas processing [4, 5] and storage [6–8] and improvements in the collection of high-quality research tissues from organ donors [9, 10] have all contributed to the evolution of pancreas and islet tissue repositories that are now available to diabetes researchers. This has also been enabled by improvements in clinical islet isolation and transplantation [9, 11], live tissue slice preparation [12, 13], donor screening [14], cryopreservation [8, 15] and profiling methods, which have yielded increasingly complex datasets. Advances in data collection and analysis have expanded the number of resources available to islet and diabetes researchers. As a result, the pancreas and islet resource landscape has become very complex, with challenges that include limited uptake of tracking tools such as Research Resource Identifiers (RRIDs) [16], non-standardised reporting, variable accessibility and prohibitive costs. As islets are critical to the pathogenesis of diabetes and related conditions, it is essential to improve access to and the usability of these resources.

In this review, we aim to provide an overview of the current landscape of human pancreatic tissue resources, coordinated phenotyping efforts and data platforms that are advancing our understanding of diabetes pathogenesis. We highlight major biobanking and islet distribution programmes that focus on archived or live tissue and explore the evolution of efforts from simple tissue repositories to sophisticated phenotyping initiatives. We discuss how new information is conveyed via an expanding ecosystem of data repositories and knowledgebases, from genomics-centric platforms to integrative tools that support multi-omics and functional data analysis. Finally, we consider future perspectives, advocating for improved accessibility, usability and donor diversity, while underscoring the need for global collaboration and open science to fully realise the translational potential of human pancreas research.

## Human pancreas/islet biobanking and distribution programmes

Our understanding of human diabetes has benefited tremendously from efforts in pancreas tissue banking and islet isolation (Table 1). These efforts have focused primarily on either banked samples or the provision of live tissue, although the lines are blurring as some traditional banking programmes begin to offer live tissue and vice versa.

**Table 1:** Fixed and live tissue biobanks focusing on the pancreas and pancreatic islets

Programme	Diabetes status^a^	Live human islets	Live pancreas slices	Banked islets^b^	Banked pancreases^b^	Other fresh tissues^c^	Other banked tissues^c^	Donor metadata^d^	Access^e^
Exeter Archival Diabetes Biobank (EADB)	ND, T1D, Aab+, T2D	No	No	No	FFPE	No	No	O (limited)	On request
Quality in Organ Donation (QUOD)	ND, T1D, T2D, T3c	No	No	No	F, FFPE, RNA, EM	No	Bl, Sp, Ur, Li, Ki, He	R	https://quod.org.uk
Network for Pancreatic Organ Donors with Diabetes (nPOD)	ND, T1D, Aab+, T2D	Pilot programme	Yes	No	F, FFPE, OCT, RNA	No	Pln, nPln, Sp, Du, Th, Ad, Pl, Se	R	https://npod.org
European network of Pancreas Organ Donors (EUnPOD)	ND, T1D	No	No	No	F, FFPE, OCT, RNA	No	Unknown	Pu	On request
Integrated Islet Distribution Program (IIDP)	ND, T2D	Yes	No	F	FFPE	Duc, Pl, Se, Sp, Pln, Ac, Du	No	R	https://iidp.coh.org
Alberta Diabetes Institute (ADI) IsletCore	ND, T1D, T2D	Yes	Pilot programme	F, C, FFPE	FFPE	Bl, Ad, Ac, Sp, Du, Pln	No	O	www.isletcore.ca
Alberta Islet Distribution Program (AIDP)	ND	Yes	No	No	No	No	No	Pu	https://sites.google.com/a/ualberta.ca/alberta-islet-distribution-program/
Pisa University Hospital collection	ND, T2D	No	No	F	FFPE, OCT	No	Ad	R, Pu	On request
Diabetes Virus Detection (DiViD)	T1D	No	No	No	FFPE	No	No	Pu	On request
LiveOnNY	ND	No	No	No	FFPE	No	Du, Sp, Pln, Se, Pbmc	Pu	https://www.liveonny.org

^a^Aab+, autoantibody positive; ND, no diabetes; T1D, type 1 diabetes; T2D, type 2 diabetes

^b^C, cryopreserved; EM, glutaraldehyde fixed; F, flash frozen; FFPE, formalin-fixed paraffin-embedded; OCT, optimal cutting temperature compound; RNA, RNAlater vials

^c^Ac, acinar; Ad, adipose; Bl, blood; Du, duodenum; Duc, ductal; He, heart; Ki, kidney; Li, liver; (n)Pln, (non)pancreatic lymph nodes; Pbmc, peripheral blood mononuclear cells; Pl, plasma; Se, serum; Sp, spleen; Th, thymus; Ur, urine

^d^O, open access; Pu, on publication; R, registered users (may require approval)

^e^Contact details are available on the BioPanc ‘Biobanks Summaries’ tab (https://clekka.shinyapps.io/biopanc_donor_atlas/)

### Primarily fixed tissue repositories

The Exeter Archival Diabetes Biobank (EADB) is a post-mortem pancreas biobank established in Glasgow in the 1980s to meet the need for tissue from young, recently diagnosed individuals with type 1 diabetes [17]. Formalin-fixed paraffin-embedded (FFPE) blocks or slides were reviewed and collated from 171 donors with type 1 diabetes, 178 without diabetes, 24 with type 2 diabetes and 23 with other pancreatic diseases. This biobank was transferred to the University of Exeter in 2016 [18]. Its unique enrichment in young-onset, short-duration donors (119 donors with a diabetes duration of <1 year) has been used to address key questions in the field [19] relating to the profiles of insulitis and impacts of age at diagnosis [20], viral aetiology [21], interferon responses, immunogenicity of beta cells [22] and selective loss of small endocrine objects in type 1 diabetes [23].

In the past 20 years, large-scale projects on islet pathophysiology have been supported by the European Union, the European Federation of Pharmaceutical Industries and Associations (EFPIA), Breakthrough T1D, and the Leona M. and Harry B. Helmsley Charitable Trust [24]. These include the Innovative Medicines Initiative for Diabetes (IMIDIA), T2DSystems, the Innovative Medicines Initiative RHAPSODY (www.imi-rhapsody.eu) and INNODIA (www.innodia.eu). The majority of the pancreases handled within these broad projects were derived from brain-dead multi-organ donors [25] and processed in a single centre under standardised procedures at the University Hospital of Pisa, where a collection of pancreatic samples from 772 donors (144 with type 2 diabetes) is available on request for collaborative initiatives [26–28].

The UK Quality in Organ Donation whole pancreas tissue bank (QUOD-PANC) was established in 2017 with Medical Research Council funding as an expansion of the UK QUOD biobank (https://quod.org.uk), which collects clinical data, blood samples and tissue biopsies from organ donors (>135,000 samples in total). Pancreases are dissected into eight anatomical regions with multimodal biopsies from each region. The QUOD-PANC Core performs standardised histology on blocks from each region, and collates data in an Atlas portal that is available with registration (https://quod.org.uk/tag/panc/). This resource has contributed insights into the pancreas in cystic fibrosis [29, 30]. The QUOD-PANC tissue bank currently comprises >140 donors (age range 6–81 years), including a majority of donors without known diabetes (HbA_1c_ during terminal admission <42 mmol/mol [6.0%]), >50 with type 2 diabetes, and ten with type 1 diabetes. Expansion of this resource will include pancreatic resection tissue from donors with chronic pancreatitis and pancreatic neoplasia.

The Network for Pancreatic Organ Donors with Diabetes (nPOD; https://npod.org), located in Gainesville, USA, was created in 2007 with the goal of obtaining pancreases, spleen, non-pancreatic lymph nodes from the mesenteric or inguinal areas, pancreatic lymph nodes, duodenum, peripheral blood, thymus and bone marrow from donors with type 1 diabetes or islet autoantibodies (Aab+) [10, 31, 32]. It was an early pioneer in this area, with many believing that such a programme was not only unfeasible but also of limited interest and would perhaps merely replicate existing knowledge gleaned from animal models. nPOD obtains transplant-grade pancreases from across the USA and has collated samples from 56 organ procurement organisations and >700 donors, making it the field’s largest biobank. nPOD also has tissue from donors with monogenic diabetes, cystic fibrosis-related diabetes, type 2 diabetes and gestational diabetes and from non-diabetic autoantibody-negative (Aab–) individuals across a range of ages [33]. Recently, nPOD added >80 donors from the Human Atlas of Neonatal Development and Early Life Pancreas (HANDEL-P). nPOD also provides live pancreas slices [12], enabling in situ study of islet/pancreas function and immune interactions [34, 35]. nPOD West at City of Hope (Los Angeles, USA) processes pancreases for simultaneous islet isolation and tissue slices. nPOD research has been recently restructured around specific ‘key questions’: (1) possible differences between type 1 diabetes in children and adults; (2) prohormone processing defects in type 1 diabetes; (3) the exocrine pancreas in type 1 diabetes development; (4) translational studies to advance type 1 diabetes therapies; (5) beta- and/or islet-specific targets; and (6) the heterogeneity of type 1 diabetes. nPOD has made an immense contribution to our understanding of the pathogenesis of type 1 diabetes [36], including key insights into the role of viruses in type 1 diabetes by the nPOD-Virus group [37–41].

The LiveOnNY organ procurement organisation (https://www.liveonny.org) maintains a biobank of pancreases from ~300 donors (and growing) with a broad age range (0–87 years) in FFPE blocks from the organ head, body and tail. Samples of adjacent spleen, pancreatic lymph nodes, immune cell suspensions and plasma are also available for a subset of donors, making this resource potentially valuable for immunology-focused studies.

Finally, it is important to note that other smaller biobanks have been, and remain, important sources of pancreas tissue and insights. This is particularly notable given the rarity of some of the samples they house. The Diabetes Virus Detection (DiViD) resource includes pancreas tail biopsies from six adults within 3–9 weeks of diagnosis and is maintained at the University of Oslo, Norway [42]. The European network of Pancreatic Organ Donors with Diabetes (EUnPOD), part of INNODIA, collected samples from eight donors according to nPOD protocols, including two with type 1 diabetes; this resource is housed at the University of Siena, Italy.

**Fig. 1:**
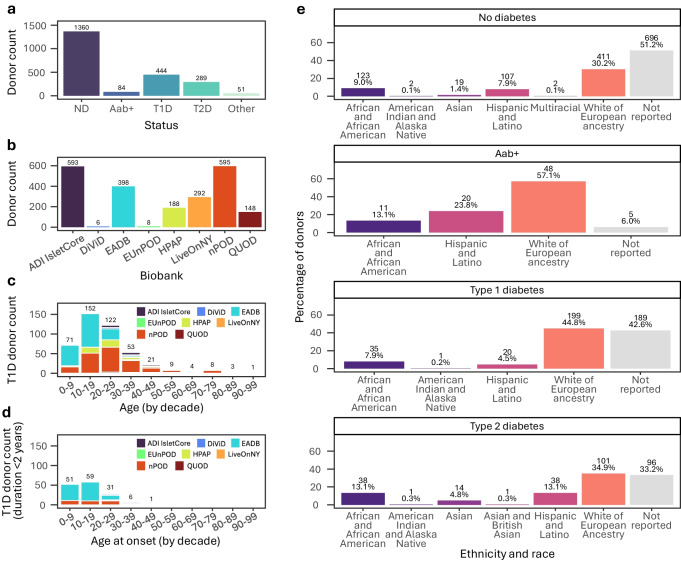
Summary of human pancreas biobanks included in the BioPanc Donor Atlas (https://clekka.shinyapps.io/biopanc_donor_atlas/). (**a**) Total donor count by diabetes status, including donors without diabetes and those with related conditions. (**b**) Total donor count per pancreas biobank. (**c**) Total type 1 diabetes donor count by age (decade) and pancreas biobank. (**d**) Total type 1 diabetes donor count by age at onset (decade) among those with diabetes of <2 years’ duration. (**e**) Percentages of donors by diabetes status and race and ethnicity. Aab+, autoantibody positive; ADI, Alberta Diabetes Institute; HPAP, Human Pancreas Analysis Program; ND, no diabetes; T1D, type 1 diabetes; T2D, type 2 diabetes. This figure is available as part of a downloadable slideset

Despite the growing importance and size of these biobanks worldwide, finding and understanding what is available in these resources can be challenging. In this regard, the BioPanc Donor Atlas (https://clekka.shinyapps.io/biopanc_donor_atlas/) outlines the availability of banked samples from >2200 donors across eight tissue banks and enables users to explore across different biobanks, filtering by donor characteristics and disease status (Fig. 1). This analysis of combined data from global collections indicates that certain donor demographics are well represented, such as type 1 diabetes donors diagnosed between ages 10 and 30 years. However, other donors are comparatively scarce, notably donors diagnosed with type 1 diabetes aged <10 years, those with a short disease duration and, especially, those diagnosed at age >30 years. Type 1 diabetes diagnosed in young children is increasingly being recognised as having distinct clinical, pathological and progression features, making it crucial that this demographic is not understudied in the field. There are currently only 15 samples from donors diagnosed with type 1 diabetes after the age of 30 years; this may be because some cases are misdiagnosed as type 2 diabetes [43].

### Primarily live islet and pancreas resources

A review of global contributors to human research islet distribution was published in 2019 [44], although it is difficult to determine the current activity of some programmes. Here, we do not discuss all individual programmes but instead describe the evolution of larger human islet isolation and distribution programmes in North America and Europe (Table 1), while recognising the potential for growth in islet distribution networks in China [45], India [46], South America [47], the Middle East [48, 49] and Australasia [50], and the unmet need for growth in Africa [51].

The Integrated Islet Distribution Program (IIDP; https://iidp.coh.org), which originated as the Islet Cell Resource in 2001 [9, 52], stems from early efforts in transplantation [53] and the refinement of human islet isolation [54], particularly via development of the Ricordi chamber [5, 55]. These advances broadened the use of human islets in research through the 1990s and 2000s, fuelled in part by an expansion of clinical islet transplant initiatives following the publication of the Edmonton Protocol [56]. The history of the IIDP and its pioneering coordination of research islet distribution is recounted elsewhere [57]. This programme has contributed greatly to the development and assessment of algorithms [58, 59] and the standardisation [60, 61] of the distribution of islets from >2500 donors across a user network. The IIDP has also enriched our understanding of variability in islet isolation outcomes across centres [62, 63]. The IIDP coordinates the distribution of human islets from donors without diabetes and with type 2 diabetes for research from ten centres across the USA and also provides other tissues, including spleen, pancreatic lymph nodes, duodenum, blood (serum and plasma), ductal and acinar samples. One of these centres, Prodo Laboratories (www.prodolabs.com), also serves as a key commercial source for human research islets and supplies other industry vendors, such as InSphero (www.insphero.com), which generates and distributes human pseudoislet products. The IIDP is notable in its strong online resources detailing both its support of the research community (>300 million islet equivalents and >1.6 million snap-frozen islets distributed since inception) and the resulting scientific publications. The IIDP has also made significant strides in islet phenotyping and data accessibility, which are described below.

Studies in Europe with live human islets started in the early 1980s [64, 65], and European Union-supported programmes have enabled prolific collaborations between several centres [66–70]. The standardisation of procedures and data analyses have been facilitated by several regional collaborations between clinical islet transplant centres, such as those in the European Collaborative Islet Transplant Registry (www.citregistry.org), the GRAGIL network [71], the Nordic Network for Islet Transplantation (https://nordicislets.medscinet.com) and the European Pancreas and Islet Transplantation Registry (EPITR; esot.org/epitr/). These networks may also provide islets for research; for example, the Nordic Network for Islet Transplantation distributes islets and other tissues from Uppsala throughout the Nordic countries [72]. Other centres also contribute to collaborative research activities beyond transplantation, with different degrees of involvement, sometimes under specific human tissue transfer agreements [66, 73–75].

In recent years, emerging regulatory differences among European nations have renewed interest in the use of surgical samples derived from pancreatectomised individuals [76–78]. While isolating whole islets from such samples remains elusive, they have been used to study insulin release from tissue slices and the molecular features of islet cells isolated by laser capture microdissection [79–81]. Surgical samples have the benefit of minimal cold ischaemia time and enable in vivo assessment of insulin secretion and insulin sensitivity before and, in some cases, after the procedure. Limitations include the possible presence of impaired glucose tolerance or diabetes due to the underlying exocrine pancreas disorder (type 3c diabetes, which may be misclassified as type 2 diabetes), and the potential influence of tumours on neighbouring cells. An inventory of European centres applying these approaches, and any associated repositories, is currently missing.

From the 1980s, efforts in Edmonton, Canada, focused on advancing human islet isolation and cryopreservation [6, 82–84] to optimise transplantation [85–88]. This culminated in the successful Edmonton Protocol for islet transplants in type 1 diabetes [11, 56, 89]. An important offshoot has been the Alberta Islet Distribution Program (AIDP; sites.google.com/a/ualberta.ca/alberta-islet-distribution-program/). This programme has performed >2500 human islet isolations (likely to be the most in the world by a single isolation centre) at the Clinical Islet Laboratory of the University of Alberta and has supported foundational science since 2007 [90, 91]. In 2010, the parallel Alberta Diabetes Institute (ADI) IsletCore (www.isletcore.ca) was established at the ADI, which, from its conception, has uniquely focused on processing research-consented donor pancreases that are not accepted for clinical whole pancreas or islet transplantation [92]. This programme, like the IIDP, makes all standard operating procedures and protocols publicly available online. In addition to freshly isolated islets from donors with and without diabetes, ADI IsletCore provides acinar, pancreatic lymph node, spleen, intestine, adipose and blood tissues, along with banked FFPE biopsies, cryopreserved islets and snap-frozen islets from >615 donors.

## Phenotyping, data repositories and knowledge bases

### Making use of tissue resources: phenotyping

A goal of our field is to understand islets well enough to (1) design treatments that correct defects in diabetes; (2) devise ways to protect islets from stresses associated with diabetes and transplantation; and (3) produce surrogates for diabetes cell therapy. For decades, most experiments involving human islets had low statistical power and often simply sought to confirm results from cell lines or rodent models. However, a hallmark feature of human islets is their variability in responsiveness [93]. This means that much of the previous work using human islets cannot stand on its own. Phenotyping efforts aim to address this by generating extensive datasets that capture and leverage the significant variability in human islet functional and molecular profiles (Table 2).

**Table 2:** Large-scale human pancreas and islet phenotyping programmes

Programme	Islet source	Tissue level(s)	Breadth	Depth of data	Data access^a^	Strengths	Website	Key publication(s)
Human Pancreas Analysis Program (HPAP)	University of Pennsylvania and Vanderbilt University, screened by nPOD	Pancreas, islets, single cell	~200 donors	Comprehensive	Or	Deepest data, freely available	https://hpap.pmacs.upenn.edu	[108, 109]
Human Islet Phenotyping Program (HIPP)	IIDP	Islets	~660 donors	Baseline	A	Strong user interface Extensive donor data Data on islets distributed to users	https://iidp.coh.org	[57]
HumanIslets consortium	ADI IsletCore	Islets, single cell	~620 donors	Intermediate	O	Simple data access and analysis tools for all experience levels	www.humanislets.com	[111]
Islet Gene View	Nordic Network for Islet Transplantation	Islets	<200 donors	Baseline	C	Easy to use, includes analysis tools	https://mae.crc.med.lu.se/IsletGeneView/	[72]

^a^A, approval required; C, closed; O, Open; Or, open with registration. Note that data access may vary depending on data type. For example, even the open access programmes may indirectly link some data to closed repositories, for example in the case of genetic data

Crucially, teams with diverse and complementary technical abilities can compile well-powered datasets on hormone secretion, cellular metabolism, stress resilience, cell survival and proliferative capacity that together dictate functional beta cell mass. These ‘hard biological endpoints’ can be complemented by omics data, which are relatively abundant but often lack physiological context. Novel insights can be achieved when omics data are obtained from deeply phenotyped islets linked with key donor characteristics. For example, by combining proteomics, transcriptomics and careful phenotyping of dynamic insulin responses to nutrients, hundreds of high-confidence protein hits associated with insulin secretion and type 2 diabetes were identified [93]. In another study, systematic and standardised testing of type 2 diabetes donor islet function and ex vivo recovery led to the unexpected finding of beta cell functional plasticity and associated transcriptome signatures [66].

### Data repositories

**Fig. 2:**
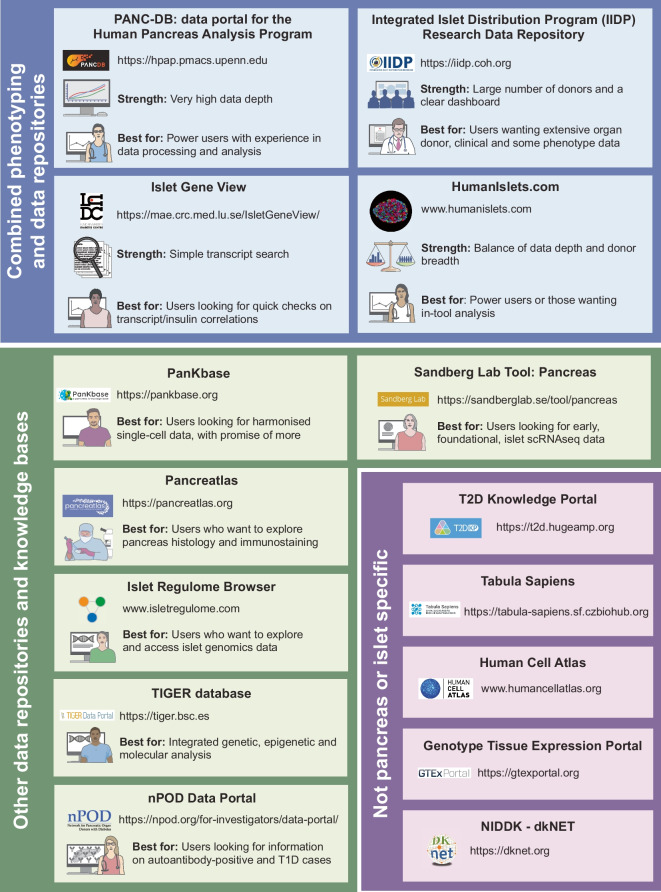
Summary of key data resources and knowledge bases. The data resources shown are linked directly to large-scale phenotyping programmes (blue), are project-based or based on data aggregation (i.e. ‘standalone’, green), or include pancreas and islet data within a larger context (purple). Key strengths and use cases are highlighted but are non-exhaustive. NIDDK, National Institute of Diabetes and Digestive and Kidney Diseases; scRNAseq, single-cell RNAseq; T1D, type 1 diabetes; T2D, type 2 diabetes. This figure is available as part of a downloadable slideset

Data repositories serve as access points for the scientific community. Generic repositories include the NCBI Gene Expression Omnibus [94] and the European Genome–phenome Archive (EGA) [95] for genomics, and ProteomeXchange [96] for proteomics. Datasets can be integrated in specialised resources using standardised and open workflows. The first islet- or pancreas-focused repositories provided access to transcriptomic and genomic datasets and included EPConDB [97], T1DBase [98] and GeneSpeed Beta Cell [99]. The focus on these types of data was influenced by the early adoption of open data principles and the establishment of minimum information guidelines in the DNA array and sequencing communities, as well as methodological developments that made the technologies fast, cheap and scalable. Most contemporary genomics resources (Fig. 2), such as the Type 2 Diabetes Knowledge Portal [100], maintain a focus on these technologies but are increasingly expanding by integrating genome-wide association study (GWAS) data and islet and donor phenotyping data. The Human Islet Research Network (HIRN) Resource Browser (https://resourcebrowser.hirnetwork.org) provides links to more than 400 human islet datasets, including omics and imaging data. The National Institute of Diabetes and Digestive and Kidney Diseases dkNET platform (https://dknet.org) serves as a centralised hub of material, computational and data resources [101] that include ‘high value data sets’ related to diabetes, including many described in the present review. Of note, the important contributions of dkNET have included promoting the use of RRIDs for resource identification.

The Islet Regulome Browser (www.isletregulome.com) allows exploration of islet-specific epigenome and transcriptome data, providing access to a wealth of information on different classes of regulatory elements, enhancer clusters, transcription factor binding sites and GWAS variants in adult human islets and pancreatic progenitors [102]. The browser can be searched for gene names or genome coordinates, together with a selection of specific chromatin maps, and the interactive interface displays virtual 4C maps, loci of type 2 diabetes SNPs, and different types of regulatory elements and transcription factor binding sites.

Pancreatlas (pancreatlas.org) was developed to share histological and immunostaining data [103]. Collections include cystic fibrosis-related diabetes samples [104], Human Pancreas Analysis Program (HPAP) samples, neonatal development and early life samples (HANDEL-P), the IIDP Human Islet Phenotyping Program (HIPP) samples, and images from the EADB [18]. Images, numbering more than 4300, can be opened directly in PathViewer (v.3.11.0, Glencoe Software, www.glencoesoftware.com) to enable visualisation, marker comparison and annotation of regions of interest. Detailed image analysis, however, requires the images to be downloaded and analysed using a specialised workflow. Raw images are not available, and users must rely on PathViewer exports, thus limiting this repository’s utility. Pancreatlas currently includes some single-cell datasets, which may position this resource well to handle and analyse future spatial omics datasets.

Histological data are also available online via the nPOD Data Portal (https://portal.jdrfnpod.org/), following an application for access. This site provides histopathology assessments based on whole slide scans from H&E-stained sections; sections stained for insulin, glucagon, somatostatin and pancreatic polypeptide to visualise endocrine cells; and sections stained with Ki67 and CD3 for quantification of cell proliferation and immune cell infiltration. These data are accessible via the Aperio eSlide Manager (https://aperioeslide.ahc.ufl.edu/), which offers similar utility and limitations to Pancreatlas. An advantage of nPOD, however, is its primary role as a tissue repository, allowing online histopathology to be used as a screening tool for selecting donors and samples for further analysis. The nPOD Data Portal also hosts donor and diabetes-related information (similar to the IIDP), along with published and unpublished datasets. These include high-resolution four-digit HLA genotypes, immune phenotyping, some functional data on live pancreas slices, custom SNP microarray donor genotyping, and a standardised whole-exome sequencing (WES) pipeline [105].

### Integrated phenotyping and data repositories

Some resources directly connect pancreas and islet phenotyping efforts to data access and/or analysis tools (Table 2). Islet Gene View (https://mae.crc.med.lu.se/IsletGeneView/) leverages phenotyping and omics output for added insight [72]. This programme from the Nordic Network for Islet Transplantation and the Human Tissue Laboratory at the Lund University Diabetes Centre (LUDC) integrates donor phenotypes, bulk RNAseq data and insulin secretion data (stimulation index) from 188 donors, along with tissue expression and islet expression quantitative trait loci (eQTL) data. The web tool allows easy searching of transcript-level correlations (e.g. correlating an islet transcript with donor phenotype), co-expression analysis, and tissue and cell type specificity (although the last uses an older, relatively small, single-cell RNAseq dataset [106]). However, while the open web tool provides easily interpretable outputs, the underlying data are not accessible, and results (or visualisations) are not downloadable.

The IIDP has made significant strides in collecting and presenting phenotyping data, enhancing the accessibility of donor information through the IIDP HIPP and the Human Islet Genotyping Initiative (HIGI), and securing an agreement to access United Network for Organ Sharing (UNOS) data. These data from >1700 human islet isolations are accessed via the IIDP Research Data Repository (https://iidp.coh.org/). Access requires an application and provides more donor-level data than other resources. Beyond age, sex and BMI, the details available include donor social and clinical history, prior treatments and medications, and information on organ processing. Available phenotyping data (~660 isolations) include measures of insulin and glucagon secretion (as stimulation index or AUC), insulin content, cell composition and cell viability. Data also include >1400 images from FFPE samples, with H&E and immunohistochemical staining for insulin and glucagon and unstained slides available on request. Approximately 360 of these donors have also been genotyped and, while genotyping data are subject to restricted access through the EGA, the web tool provides polygenic risk scores for both type 1 and type 2 diabetes for these donors along with genetic ancestry [107]. Strengths of the IIDP Research Data Repository include an easy-to-use web dashboard, extensive pancreas/islet donor information, which increasingly includes genetic risk assessment, and islet phenotyping data from a relatively large number of donors. The available data are integrated across isolation, donor, phenotype and genotype results; however, the resource provides no tools for data analysis.

The HPAP seeks to phenotype pancreases from Aab+ organ donors, donors with early type 1 diabetes and donors with type 2 diabetes, compared with reasonably matched control individuals [108, 109]. It is worth noting that many adult donors who are positive for a single autoantibody may not progress to type 1 diabetes. Processing pancreases from donors identified through a collaboration with nPOD, this programme undertakes what can be considered the most comprehensive pancreas and islet phenotyping currently available. To date, the HPAP has profiled pancreas samples from 193 donors (22 Aab+, 45 with type 1 diabetes, 52 with type 2 diabetes), although these are used for extensive in-house profiling and are not distributed directly to the research community. In addition to standard donor characteristics data, the associated PANC-DB data portal (https://hpap.pmacs.upenn.edu) provides bulk islet and single-cell genomics, whole-genome sequencing, dynamic hormone secretion and oxygen consumption data, and spatial data (standard histology, CODEX/PhenoCycler, imaging mass cytometry). A strength of the HPAP and the PANC-DB data portal is the depth of phenotyping data that is freely and quickly available via a simple, free registration. However, the ability to analyse this data can require specialised expertise, and some datasets (e.g. Ca^2+^ imaging) may therefore be underused. A more user-friendly web tool has been developed to enable gene expression analysis in single islet cells from HPAP donors, allowing subsets of donors with specific demographic (age, sex, ethnicity) or clinical characteristics (Aab+, type 1 diabetes, type 2 diabetes, no diabetes, disease duration, HbA_1c_, BMI) to be selected [110].

The HumanIslets consortium (www.humanislets.com) focuses on islets and pancreases processed by the ADI IsletCore [111], leveraging a broad islet distribution network and decentralised phenotyping linked back to the same donors. With data from 588 donors currently available on this platform, it is among the broadest of such resources. Although phenotyping is not as comprehensive as in the HPAP, several data types are unique, including whole-islet proteomics, electrophysiology, dynamic insulin responses to nutrients beyond glucose, and prohormone processing [93, 112, 113]. A strength of the HumanIslets consortium is its accessibility to biologists without specialised bioinformatics skills, enabling user-friendly analyses and gene/protein lookups directly on the website. The underlying data, metadata, analysis results and associated workflows can be downloaded to enable more flexible or customised analysis. For bulk RNAseq data, the HumanIslets consortium provides a highly accessible dataset, offering users raw counts, TPM (transcripts per kilobase million) and logCPM (log-transformed counts per million) data following batch correction in a spreadsheet format with clear documentation. Advanced bioinformatics experts seeking to work with the raw data can follow links to the relevant repositories.

### Aggregating data for power and insight

The open access Translational human pancreatic Islet Genotype tissue-Expression Resource (TIGER) portal compiled data on >500 human islet preparations (including 30 from type 2 diabetes donors) from five cohorts. Bulk RNAseq and array-based genotypes underwent stringent quality control, and genotype imputation using four different reference panels improved coverage of low-frequency and rare variants that may have larger effect sizes on gene expression and diabetes risk [68]. Genetic variation was associated with gene expression by eQTL studies in four islet cohorts and subsequently meta-analysed, resulting in >1 million eQTLs. The user-friendly TIGER browser enables users to look up gene expression, (epi)genomic regions, eQTL and allele-specific expression, with gene- or genetic variant-centric queries, and provides links to external resources (i.e. UCSC Genome Browser, Gene Ontology [GO] terms, Ensembl and UniProtKB/Swiss-Prot). Colocalisation of GWAS and eQTL SNPs permits risk variants for glycaemic traits, type 1 diabetes and type 2 diabetes to be linked with islet gene expression [68, 114]. TIGER thereby provides insight into the molecular underpinnings of islet pathophysiology and the genetics of diabetes.

The Pancreas Knowledge Base (PanKbase; pankbase.org), started in 2024, is a centralised resource for human islet and pancreas data. Its goal is to aggregate datasets and curate knowledge from multiple sources to generate robust, standardised and computationally ready omics data, analytic libraries and pipelines, and metadata. Available resources currently include single-cell RNAseq and single-nucleus ATAC-seq data from islets obtained through the HPAP and IIDP programmes, as well as a commercial source (Prodo Laboratories). This is the largest single-cell map to date, processed through a harmonised pipeline. The data can be downloaded, explored and examined using an integrated cell browser. Tools for principal component analysis (PCA) and cell-specific pseudo-bulk differential expression analysis are also available. A particularly novel component of PanKbase is the pancreas knowledge graph (PanKgraph), a prompt-based artificial intelligence (AI)-guided knowledge repository that enables the exploration of links between transcript expression and SNPs, with a primary focus on type 1 diabetes. PanKbase is a relatively new resource and is currently under active development. With a stated commitment to the Findable, Accessible, Interoperable, and Reusable (FAIR) principles of data curation (www.go-fair.org), this promises to be a key resource for curating and accessing datasets from disparate sources.

## Challenges and gaps

In contrast to studies on inbred rodents, human islet data are shaped by more complex factors that can influence molecular and functional phenotypes. Donor metadata such as age, BMI, HbA_1c_ and sex associate with islet cell composition [115, 116], insulin content [92, 117] and insulin secretion [92, 117–119]. Nearly 10 years ago, this field was encouraged to improve transparency in the description of human islets used in research [120]. In response, several journals, including *Diabetologia*, called for the reporting of (limited) donor characteristics [121]. While a welcome advance, the availability of donor characteristics remains a common limitation, with factors such as genetic ancestry, menopause status and diabetes duration potentially influencing molecular and functional outcomes. This gap may be bridged through studies of islets from live donors [69], although existing gaps in representation across ages and ethnicities (Fig. 1) may continue. In addition, metadata may be incorrect; the reported donor sex was incorrect in 3.7% of TIGER samples based on Y chromosome gene expression. Metadata reporting may be improved through the greater uptake of RRIDs, although these are not the ultimate solution, as the parameters reported at best explain only a minor proportion of the variation in islet isolation [63, 122]. In addition to clinical data, technical parameters such as purity, cold ischaemia time and islet culture duration also affect islet insulin content and secretory function [92, 117]. Understanding the technical and biological factors that drive heterogeneity in islet isolation outcomes and islet function remains an important question.

### Variability and standardisation

**Fig. 3:**
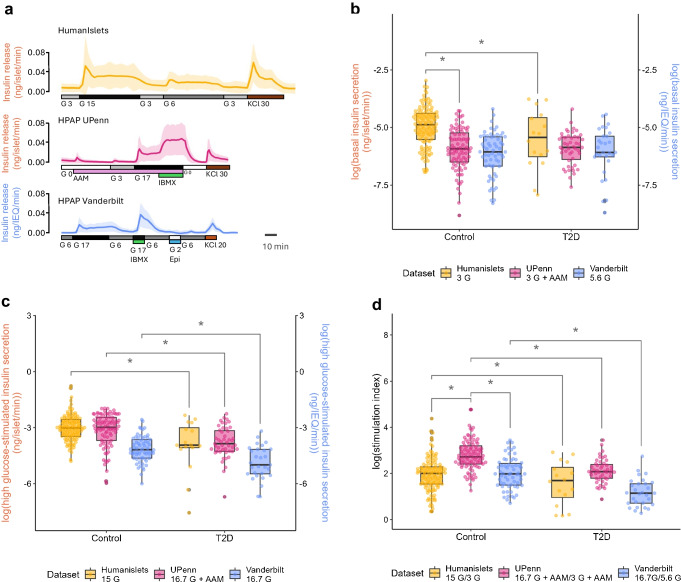
Comparison of glucose-stimulated dynamic insulin secretion from different centres. (**a**) Dynamic insulin secretion measurements from HumanIslets.com, performed at the University of British Columbia, or from the HPAP, performed at the University of Pennsylvania (UPenn) or Vanderbilt University. Data from HumanIslets.com and UPenn are normalised per islet, while data from Vanderbilt are normalised per islet equivalent (IEQ). (**b**–**d**) Summary of mean basal insulin secretion (**b**), maximum high glucose-stimulated insulin secretion (**c**) and the simulation index, calculated as high glucose-stimulated insulin secretion over basal insulin secretion (**d**). Note the different conditions used at each centre. *p<0.05 by two-way ANOVA with Tukey’s correction. For basal and high glucose-stimulated secretion, comparisons between the Vanderbilt University dataset and the other two datasets were not performed because of the different normalisation methods used. AAM, amino acid mix; Epi, adrenaline (epinephrine); G, glucose; IBMX, isobutylmethylxanthine; T2D, type 2 diabetes. This figure is available as part of a downloadable slideset

Islet phenotyping consortia developed organically and independently of each other, typically around major islet isolation centres. As a result, experimental conditions are not standardised, making it challenging to pool data across centres, despite recent efforts (e.g. many centres use and recommend Prodo media, although the composition of the different types of media is not disclosed). A consistent feature of human islet data is significant variation among donors [93], which can limit our ability to resolve key biological differences. Stimulation indices measured during dynamic insulin secretion assays show coefficients of variance of ~95% (calculated from data at HumanIslets.com and PANC-DB). This means that detecting a 20% difference between two groups at 80% power and α=0.05 requires a sample size of ~370. Such sample sizes are currently not achievable with a single data source, precluding comparison of minority donor subgroups, such as female donors (40–46% of total donors), donors with diabetes (3–23% type 1 diabetes, 11–27% type 2 diabetes), donors of specific age brackets (12–46% aged <40 years) and donors from minority ancestry groups (<24%), donors with BMI <25 kg/m^2^ (~35%), or donors with multiple such characteristics. One solution is to combine data from several sources, which has been accomplished with RNAseq [123–126]. However, other data types are frequently obtained using site-specific protocols, making their combination challenging, if not impossible. Publicly available data on dynamic insulin secretion [57, 108, 111] are generated using different stimuli, timings, flow rates, normalisation modes (number of islets vs islet equivalents), lysis solutions for extracting insulin content, and assay kits. These differences are non-trivial (Fig. 3). Looking ahead to improving current programmes and establishing new centres, with the benefit of hindsight, it would be incredibly powerful to synchronise at least some experimental protocols for procuring functional data.

One of the largest sources of between-consortia variation may be the decision to hand-pick islets after shipment. While hand-picking islets removes damaged or fragmented islets and exocrine tissue from even high-purity preparations, potentially reducing exposure to enzymes that stress islets, this process may select for uniformity of islet size (ignoring small islets) that may not accurately reflect the in vivo situation. As a result, stimulation indices for static incubation and perifusion studies from groups that hand-pick can be at least twofold greater than those from groups that do not [122]. Even within consortia, potential sources of variation persist that could benefit from standardisation. For example, circadian variation in gene expression [127] and metabolism [128] suggests important impacts that could be addressed by introducing synchronisation protocols or, at the very least, by recording the time of experiments.

Clear and methodical data and metadata reporting can improve accessibility and provide structure to support future standardisation across data sources. Ideally, a user should be able to download, understand and analyse a dataset without needing to contact the data manager for clarification. Ideal metadata reporting should be intuitive while conforming to local and regional laws governing patient privacy, with clear data headings. For example, the HPAP and IIDP provide detailed metadata files with logically ordered sheets, consistent use of RRIDs and, in the case of the IIDP, a clear distinction between ethnicity and race/ancestry. Data reporting should be accompanied by protocols that are sufficiently detailed for the user to interpret the data, including updates as needed, and easy to locate in the user interface. For example, HumanIslets.com has a dedicated documentation section that provides such details. This is particularly critical when only raw data files are provided and methodological details are required for proper data processing.

### Gaps in geographical and donor diversity

Genetic ancestry has important influences on diabetes risk and presentation, including islet characteristics. First, we note that different resources assess and define ancestry, race and ethnicity in different ways, whether through genetic testing or self-reporting. Here, we use terminology as recommended in guidelines from *JAMA* [129]. Isolated islet perifusion data from the HPAP show a greater type 2 diabetes-associated decrease in insulin secretion among donors of Black/African American ethnicity than among White donors of European ancestry [130], and substantial variation in insulin secretion across genetic ancestry groups [119]. A recent study found that a distinct type 1 diabetes subtype in which autoantibodies are absent is common among sub-Saharan African individuals, a finding also observed among Black participants in the US SEARCH cohort [131]. Overlooking the effects of ancestry and geography on diabetes risk and progression may limit the efficacy of treatment and management strategies. While current islets datasets provide some insight, these remain heavily weighted towards individuals of European ancestry.

Globally, human islet cores are concentrated in Europe and North America, with a handful in South America, Australia, East Asia and Singapore [44]. Even in countries such as the USA and disregarding the crude classification of heterogeneous ethnic groups [132, 133], the donor pool shows under-representation of diverse racial and ethnic groups, among whom diabetes onset, progression and complications vary [134, 135]. Indeed, when excluding samples from donors where ancestry is not reported, most of the tissue in biobanks derives from White donors of European ancestry (61.9%). Among type 1 diabetes donors, this proportion is higher (78.0%), while it is lower among type 2 diabetes donors (52.3%) (Fig. 1e). Consideration of genetic ancestry in islet phenotyping efforts remains an important challenge.

Assembling tissues across the life course is also important. Initiatives such as HANDEL-P and the Human Developmental Biology Resource are facilitating studies in young donors, providing insights into the early stages of pancreatic development. In summary, the remarkable work of organ procurement organisations, biobanks and global researchers is significantly expanding our understanding of the human pancreas. However, given limited donor representation, we must exercise caution before generalising findings.

### Barriers to data access

While the resources mentioned above include abundant data, accessibility remains an area for improvement. Cross-cutting issues include experimental protocols with limited or outdated information, unclear (or absent) data preprocessing, and data downloads that are either unavailable or pose unnecessary barriers for laboratories without a strong computational focus. The rapid expansion of multi-omics and phenotyping technologies has created a huge volume of complex, high-dimensional datasets, but post hoc analyses remain limited by inconsistencies in cell type annotations and the availability of processed count data [136]. To extract meaningful insights, standardised reporting, principled methods of access and dedicated analytical pipelines are essential to fully capitalise on the substantial investments in human islet research.

It is, however, important to exercise care when performing post hoc analyses of already processed data, given the potential confounding factors discussed above in ‘Variability and standardisation’. Indeed, naive integration may obscure disease-specific differences that are later brought to light via improvements in dataset integration [137, 138]. Thus, inconsistent systematic and programmatic data availability remains a major bottleneck. In particular, access to raw data and standardised metadata is important as it enables future reanalysis using improved analytical pipelines and facilitates improved integrative analyses that are not confounded by differences in upstream data processing. Metadata standards for reporting islet characteristics exist [120, 121], but they require additional specification and definition using controlled ontologies and increased adaptation in the field. For example, which units are HbA_1c_ reported in? Which clinical definition was used to diagnose diabetes? It is essential for the community to establish and maintain reporting standards that incorporate information about treatments. In addition to incoherent metadata reporting, the datasets themselves are scattered across multiple public and private databases with inconsistent structures and metadata requirements, making data collection and curation cumbersome and time consuming. A solution to this problem is to create secondary databases that collect and curate data from a diverse set of primary databases and share it in accordance with FAIR principles. Efforts such as Type 2 Diabetes Knowledge Portal and PanKbase are working to bring resources together, but often provide access only to processed data summaries. Data protection and privacy regulations create barriers to sharing genotypes and raw sequencing data. While these safeguards are necessary, they slow/hinder data access, partly due to differences in the interpretation and implementation of the General Data Protection Regulation (GDPR) across the European Union [139]. Solutions, inspired by experiences from large international consortia, such as the Human Cell Atlas (https://humancellatlas.org), include defining a code of conduct for data sharing that details a standardised privacy-compliant data use agreement, donor consent templates, and data sharing standards that assess identifiability while maintaining FAIR and open data standards.

### Data usability and analytics

Data usability is an important bottleneck when analyses require bioinformatics skills or proprietary software. Lowering the barriers to accessing, analysing and exploring data through point-and-click interfaces can accelerate discovery. Resources such as HumanIslets.com and PanKbase integrate analytical tools by co-locating data and analytics. These initial initiatives are currently restricted to basic analyses with limited flexibility. As data collection evolves to include more omics and imaging modalities, simply providing data access will be insufficient. Future data repositories should integrate data analytics to maximise data reuse.

Multi-omics integration methods fall into two main categories: knowledge driven and data driven. In knowledge-driven approaches, researchers first analyse each individual omics dataset to identify their molecular signatures, which are then mapped onto a pre-existing knowledge base of molecular relationships, such as gene regulatory networks, protein–protein interaction networks or metabolic pathways. Researchers visualise and interpret how different omics layers interact to reveal underlying biological processes [140]. Data-driven approaches integrate multiple omics datasets using statistical or machine learning techniques such as multivariate correlation analysis or dimensionality reduction to identify shared patterns across omics layers [141]. These approaches can be used together [141]. A significant limitation of current multi-omics integration is the insufficient support for complex metadata. While some tools allow researchers to visualise how different experimental factors affect the results, most do not formally incorporate this metadata into the analytical model.

Multimodal analysis, integrating spatiotemporal maps of molecules such as genes, proteins and metabolites, allows researchers to link gene expression, protein abundance and other molecular data to their exact locations within a tissue [142]. This enables detailed reconstruction and dynamic monitoring of complex biological microenvironments, such as the islet. By overlaying molecular data onto high-resolution histology or immunostaining images, researchers can visually select regions of interest, then perform targeted analyses on these regions to identify key molecules and biological functions. The development of efficient and intuitive data analysis and visualisation tools will be critical.

AI is increasingly vital for data analysis and interpretation, particularly in complex fields such as biology. While deep learning models are powerful for tasks such as multi-omics integration and image recognition, they often act as a ‘black box’, making their decisions difficult to interpret compared with traditional methods. The rise of generative AI, such as large language models and knowledge graphs, offers a promising solution. Integrating these technologies into current knowledge bases and computational workflows will help make AI-driven insights more interpretable and greatly democratise data analysis to accelerate knowledge, discovery and translation.

## Looking forward

We require deep sampling across data modalities and donor diversity for system-level insights into islets in health and disease. Demographic imbalances limit the generalisability of current resources. Organ donor data typically provide a single snapshot of disease, often at end-stage or after years of progression, but do not capture longitudinal trajectories. Thus, it is important to continue methodological developments to enable scalable data generation for dense sampling across demographic groups and disease stages. We envision that new insights will be driven not only by the integration of additional datasets and modalities into knowledge bases, but also by technological advancements in information retrieval and analysis. An interesting avenue to explore will be the creation of scientific reasoning engines using an appropriately tuned large language model that has access to the knowledge bases enhanced with biologically informed knowledge graphs, which, through graph-based retrieval-augmented generation, can aid researchers in discovering new associations by linking disparate pieces of information through multi-hop retrieval across biologically plausible associations.

The experience and expertise gathered by established phenotyping programmes should be leveraged to support the development of similar islet distribution centres and phenotyping programmes in regions that lack such resources, building a global foundation for islet research that includes researchers, clinicians, donors and their families, and beneficiaries of all backgrounds. A key opportunity lies with human islet isolation programmes in regions that are not connected to broader phenotyping initiatives but that may be ideally poised to collaborate with existing programmes. Such collaborations will critically address the diversity and ancestry gaps in our understanding of human islet variability, enable consolidation of protocols and the development of clear, standardised reporting mechanisms to support powerful phenotyping data generation, and support extraction of the most useful possible data from precious donor samples.

## Supplementary Information

Below is the link to the electronic supplementary material.

Slideset of figures (PPTX 659 KB)

## Abbreviations

ADI: Alberta Diabetes Institute
AI: Artificial intelligence
EADB: Exeter Archival Diabetes Biobank
EGA: European Genome–phenome Archive
eQTL: Expression quantitative trait locus
FFPE: Formalin-fixed paraffin-embedded
HANDEL-P: Human Atlas of Neonatal Development and Early Life Pancreas
HIPP: Human Islet Phenotyping Program
HPAP: Human Pancreas Analysis Program
IIDP: Integrated Islet Distribution Program
nPOD: Network for Pancreatic Organ Donors with Diabetes
RRID: Research Resource Identifier

## References

[1] Warren S (1953) An interpretation of diabetes in the light of its pathology. Diabetes 2(4):257–261. 10.2337/diab.2.4.257 13083462 10.2337/diab.2.4.257

[2] Ferguson J, Allsopp RH, Taylor RMR, Johnston IDA (1977) Isolation of viable human pancreatic islets. World J Surg 1(1):69–75. 10.1007/bf01654736 325916 10.1007/BF01654736

[3] Ashcroft SJH, Bassett JM, Handle PJ (1971) Isolation of human pancreatic islets capable of releasing insulin and metabolising glucose in vitro. Lancet 297(7705):888–889. 10.1016/s0140-6736(71)92445-7 10.1016/s0140-6736(71)92445-7 4102029

[4] Gray DW, McShane P, Grant A, Morris PJ (1984) A method for isolation of islets of Langerhans from the human pancreas. Diabetes 33(11):1055–1061. 10.2337/diabetes.33.11.1055 6437895 10.2337/diab.33.11.1055

[5] Ricordi C, Lacy PE, Finke EH, Olack BJ, Scharp DW (1988) Automated method for isolation of human pancreatic islets. Diabetes 37(4):413–420. 10.2337/diab.37.4.413 3288530 10.2337/diab.37.4.413

[6] Kneteman NM, Rajotte RV (1985) Isolation and cryopreservation of human pancreatic islets. Life Support Syst J Eur Soc Artif Organs 3(Suppl 1):712–718 3916635

[7] Lakey JRT, Warnock GL, Ao Z, Rajotte RV (1996) Bulk cryopreservation of isolated islets of Langerhans. Cell Transplant 5(3):395–404. 10.1177/096368979600500306 8727008 10.1177/096368979600500306

[8] Manning Fox JE, Lyon J, Dai XQ et al (2015) Human islet function following 20 years of cryogenic biobanking. Diabetologia 58(7):1503–1512. 10.1007/s00125-015-3598-4 10.1007/s00125-015-3598-4 PMC4472956 25930156

[9] Kaddis JS, Olack BJ, Sowinski J, Cravens J, Contreras JL, Niland JC (2009) Human pancreatic islets and diabetes research. JAMA 301(15):1580–1587. 10.1001/jama.2009.482 19366778 10.1001/jama.2009.482 PMC3763818

[10] Campbell-Thompson M, Wasserfall C, Kaddis J et al (2012) Network for Pancreatic Organ Donors with Diabetes (nPOD): developing a tissue biobank for type 1 diabetes. Diabetes Metab Res Rev 28(7):608–617. 10.1002/dmrr.2316 10.1002/dmrr.2316 PMC3456997 22585677

[11] Shapiro AMJ, Ricordi C, Hering BJ et al (2006) International trial of the edmonton protocol for islet transplantation. New Engl J Med 355(13):1318–1330. 10.1056/nejmoa061267 17005949 10.1056/NEJMoa061267

[12] Speier S, Rupnik M (2003) A novel approach to in situ characterization of pancreatic β-cells. Pflügers Archiv 446(5):553–558. 10.1007/s00424-003-1097-9 12774232 10.1007/s00424-003-1097-9

[13] Cohrs CM, Chen C, Atkinson MA, Drotar DM, Speier S (2024) Bridging the gap: pancreas tissue slices from organ and tissue donors for the study of diabetes pathogenesis. Diabetes 73(1):11–22. 10.2337/dbi20-0018 38117999 10.2337/dbi20-0018 PMC10784654

[14] Wasserfall C, Montgomery E, Yu L et al (2016) Validation of a rapid type 1 diabetes autoantibody screening assay for community-based screening of organ donors to identify subjects at increased risk for the disease. Clin Exp Immunol 185(1):33–41. 10.1111/cei.12797 27029857 10.1111/cei.12797 PMC4908288

[15] Lakey JRT, Anderson TJ, Rajotte RV (2001) Novel approaches to cryopreservation of human pancreatic islets. Transplantation 72(6):1005–1011. 10.1097/00007890-200109270-00005 11579292 10.1097/00007890-200109270-00005

[16] Bandrowski AE, Martone ME (2016) RRIDs: a simple step toward improving reproducibility through rigor and transparency of experimental methods. Neuron 90(3):434–436. 10.1016/j.neuron.2016.04.030 27151636 10.1016/j.neuron.2016.04.030 PMC5854161

[17] Foulis AK, Liddle CN, Farquharson MA, Richmond JA, Weir RS (1986) The histopathology of the pancreas in type I (insulin-dependent) diabetes mellitus: a 25-year review of deaths in patients under 20 years of age in the United Kingdom. Diabetologia 29(5):267–274. 10.1007/bf00452061 3522324 10.1007/BF00452061

[18] Morgan NG, Richardson SJ, Powers AC, Saunders DC, Brissova M (2022) Images from the exeter archival diabetes biobank now accessible via pancreatlas. Diabetes Care 45(12):e174–e175. 10.2337/dc22-1613 36239401 10.2337/dc22-1613 PMC9862522

[19] Morgan NG, Richardson SJ (2018) Fifty years of pancreatic islet pathology in human type 1 diabetes: insights gained and progress made. Diabetologia 61(12):2499–2506. 10.1007/s00125-018-4731-y 30255378 10.1007/s00125-018-4731-y PMC6223849

[20] Leete P, Oram RA, McDonald TJ et al (2020) Studies of insulin and proinsulin in pancreas and serum support the existence of aetiopathological endotypes of type 1 diabetes associated with age at diagnosis. Diabetologia 63(6):1258–1267. 10.1007/s00125-020-05115-6 32172310 10.1007/s00125-020-05115-6 PMC7228905

[21] Richardson SJ, Willcox A, Bone AJ, Foulis AK, Morgan NG (2009) The prevalence of enteroviral capsid protein vp1 immunostaining in pancreatic islets in human type 1 diabetes. Diabetologia 52(6):1143–1151. 10.1007/s00125-009-1276-0 19266182 10.1007/s00125-009-1276-0

[22] Richardson SJ, Rodriguez-Calvo T, Gerling IC et al (2016) Islet cell hyperexpression of HLA class I antigens: a defining feature in type 1 diabetes. Diabetologia 59(11):2448–2458. 10.1007/s00125-016-4067-4 27506584 10.1007/s00125-016-4067-4 PMC5042874

[23] Murrall K, Luckett T, Lekka C et al (2025) Small things matter: lack of extra-islet beta cells in type 1 diabetes. Sci Adv 11(46):eadz2251. 10.1126/sciadv.adz2251 10.1126/sciadv.adz2251 PMC12609120 41223263

[24] Marchetti P, Schulte AM, Marselli L et al (2019) Fostering improved human islet research: a European perspective. Diabetologia 62(8):1514–1516. 10.1007/s00125-019-4911-4 31197398 10.1007/s00125-019-4911-4 PMC6647243

[25] Marchetti P, Suleiman M, Marselli L (2018) Organ donor pancreases for the study of human islet cell histology and pathophysiology: a precious and valuable resource. Diabetologia 61(4):770–774. 10.1007/s00125-018-4546-x 29354869 10.1007/s00125-018-4546-x PMC6449064

[26] Dotta F, Censini S, van Halteren AGS et al (2007) Coxsackie B4 virus infection of β cells and natural killer cell insulitis in recent-onset type 1 diabetic patients. Proc Natl Acad Sci 104(12):5115–5120. 10.1073/pnas.0700442104 17360338 10.1073/pnas.0700442104 PMC1829272

[27] Cnop M, Igoillo-Esteve M, Rai M et al (2012) Central role and mechanisms of β-cell dysfunction and death in friedreich ataxia–associated diabetes. Ann Neurol 72(6):971–982. 10.1002/ana.23698 23280845 10.1002/ana.23698 PMC4900175

[28] Cinti F, Bouchi R, Kim-Muller JY et al (2016) Evidence of β-cell dedifferentiation in human type 2 diabetes. J Clin Endocrinol Metab 101(3):1044–1054. 10.1210/jc.2015-2860 26713822 10.1210/jc.2015-2860 PMC4803182

[29] Kattner N, Hang Y, Krentz NAJ et al (2025) Identification of a vimentin-expressing α-cell phenotype in CF and normal pancreas. J Endocrinol 264(3):e240190. 10.1530/joe-24-0190 39836539 10.1530/JOE-24-0190 PMC11850051

[30] White MG, Maheshwari RR, Anderson SJ et al (2019) In situ analysis reveals that CFTR is expressed in only a small minority of β-cells in normal adult human pancreas. J Clin Endocrinol Metab 105(5):dgz209. 10.1210/clinem/dgz209 10.1210/clinem/dgz209 PMC7341165 31748811

[31] Kaddis JS, Pugliese A, Atkinson MA (2015) A run on the biobank: what have we learned about type 1 diabetes from the nPOD tissue repository? Curr Opin Endocrinol Diabetes Obes 22(4):290–295. 10.1097/med.0000000000000171 26087339 10.1097/MED.0000000000000171

[32] Pugliese A, Vendrame F, Reijonen H, Atkinson MA, Campbell-Thompson M, Burke GW (2014) New insight on human type 1 diabetes biology: nPOD and nPOD-transplantation. Curr Diabetes Rep 14(10):530. 10.1007/s11892-014-0530-0 10.1007/s11892-014-0530-0 PMC4174350 25142715

[33] Perry DJ, Shapiro MR, Chamberlain SW et al (2023) A genomic data archive from the Network for Pancreatic Organ donors with Diabetes. Sci Data 10(1):323. 10.1038/s41597-023-02244-6 37237059 10.1038/s41597-023-02244-6 PMC10219990

[34] Panzer JK, Hiller H, Cohrs CM et al (2020) Pancreas tissue slices from organ donors enable in situ analysis of type 1 diabetes pathogenesis. JCI Insight 5(8):e134525. 10.1172/jci.insight.134525 10.1172/jci.insight.134525 PMC7205437 32324170

[35] Panzer JK, Garcia PA, Pugliese A (2024) Generating human pancreatic tissue slices to study endocrine and exocrine pancreas physiology. J Vis Exp. 10.3791/66468 38557588 10.3791/66468

[36] Kusmartseva I, Posgai A, Yang M et al (2024) The human pancreas in type 1 diabetes: lessons learned from the Network of Pancreatic Organ Donors with Diabetes. Cold Spring Harb Perspect Med a041588. 10.1101/cshperspect.a041588 10.1101/cshperspect.a041588 PMC12667406 39134385

[37] Rodriguez-Calvo T, Laiho JE, Oikarinen M et al (2025) Enterovirus VP1 protein and HLA class I hyperexpression in pancreatic islet cells of organ donors with type 1 diabetes. Diabetologia 68(6):1197–1210. 10.1007/s00125-025-06384-9 40090995 10.1007/s00125-025-06384-9 PMC12069150

[38] Richardson SJ, Rodriguez-Calvo T, Laiho JE et al (2025) Joint analysis of the nPOD-Virus Group data: the association of enterovirus with type 1 diabetes is supported by multiple markers of infection in pancreas tissue. Diabetologia 68(6):1226–1241. 10.1007/s00125-025-06401-x 40090994 10.1007/s00125-025-06401-x PMC12069141

[39] Laiho JE, Oikarinen S, Morfopoulou S et al (2025) Detection of enterovirus RNA in pancreas and lymphoid tissues of organ donors with type 1 diabetes. Diabetologia 68(6):1211–1225. 10.1007/s00125-025-06359-w 40095061 10.1007/s00125-025-06359-w PMC12069483

[40] Apaolaza PS, Balcacean D, Zapardiel-Gonzalo J et al (2021) Islet expression of type I interferon response sensors is associated with immune infiltration and viral infection in type 1 diabetes. Sci Adv 7(9):eabd6527. 10.1126/sciadv.abd6527 33627420 10.1126/sciadv.abd6527 PMC7904254

[41] Laiho JE, Oikarinen M, Richardson SJ et al (2016) Relative sensitivity of immunohistochemistry, multiple reaction monitoring mass spectrometry, in situ hybridization and PCR to detect Coxsackievirus B1 in A549 cells. J Clin Virol 77:21–28. 10.1016/j.jcv.2016.01.015 26875099 10.1016/j.jcv.2016.01.015 PMC5364806

[42] Krogvold L, Edwin B, Buanes T et al (2014) Pancreatic biopsy by minimal tail resection in live adult patients at the onset of type 1 diabetes: experiences from the DiViD study. Diabetologia 57(4):841–843. 10.1007/s00125-013-3155-y 24429579 10.1007/s00125-013-3155-y

[43] Thomas NJ, Lynam AL, Hill AV et al (2019) Type 1 diabetes defined by severe insulin deficiency occurs after 30 years of age and is commonly treated as type 2 diabetes. Diabetologia 62(7):1167–1172. 10.1007/s00125-019-4863-8 30969375 10.1007/s00125-019-4863-8 PMC6559997

[44] Ng NHJ, Tan WX, Koh YX, Teo AKK (2019) Human islet isolation and distribution efforts for clinical and basic research. OBM Transplant 3(2):1–1. 10.21926/obm.transplant.1902068

[45] Liang R, Sun P, Cai X et al (2023) Characteristics of research-focused human islet preparations from organ donors with type 2 diabetes. Islets 15(1):2219104. 10.1080/19382014.2023.2219104 37314095 10.1080/19382014.2023.2219104 PMC10269413

[46] Gandasi NR, Rangarajan A, Rao H, Singh M, Kothegala L (2023) Pancreatic islet biobanking facilities in India: the need of the hour to deal with diabetes? J Indian Inst Sci 103(1):381–385. 10.1007/s41745-023-00366-9

[47] Rheinheimer J, Bauer AC, Silveiro SP et al (2015) Human pancreatic islet transplantation: an update and description of the establishment of a pancreatic islet isolation laboratory. Arch Endocrinol Metab 59(2):161–170. 10.1590/2359-3997000000030 25993680 10.1590/2359-3997000000030

[48] Azarpira N, Aghdai MH, Nikeghbalian S et al (2013) Human islet cell isolation: the initial step in an islet transplanting program in Shiraz, Southern Iran. Exp Clin Transplant Off J Middle East Soc Organ Transplant 12(2):139–142. 10.6002/ect.2012.0306 10.6002/ect.2012.0306 23477484

[49] Larijani B, Arjmand B, Amoli MM et al (2012) Establishing a cGMP pancreatic islet processing facility: the first experience in Iran. Cell Tissue Bank 13(4):569–575. 10.1007/s10561-011-9273-1 21818570 10.1007/s10561-011-9273-1

[50] Hawthorne WJ, Davies S, Mun H et al (2021) Successful islet outcomes using Australia-wide donors: A national centre experience. Metabolites 11(6):360. 10.3390/metabo11060360 10.3390/metabo11060360 PMC8229735 34198953

[51] Mohamed F, Adams C, Mansfield B (2024) An unmet need: pancreatic beta cell replacement. S Afr Méd J 114(3b):e1249–e1249. 10.7196/samj.2024.v114i3b.1249 39041449 10.7196/SAMJ.2024.v114i3b.1249

[52] Knazek RA (2002) The human pancreatic islet cell resource consortium. Diabetes Technol Ther 4(4):551–552. 10.1089/152091502760306652 12396750 10.1089/152091502760306652

[53] Lacy PE, Scharp DW (1986) Islet transplantation in treating diabetes. Annu Rev Med 37(1):33–40. 10.1146/annurev.me.37.020186.000341 3085580 10.1146/annurev.me.37.020186.000341

[54] Alderson D, Kneteman NM, Scharp DW (1987) The isolation of purified human islets of Langerhans. Transplant Proc 19(1 Pt 2):916–917 3152636

[55] Piemonti L, Pileggi A (2013) 25 years of the Ricordi automated method for islet isolation. CellR4 Repair Replace Regen Reprogram 1(1):e128 PMC6267808 30505878

[56] Shapiro AMJ, Lakey JRT, Ryan EA et al (2000) Islet transplantation in seven patients with type 1 diabetes mellitus using a glucocorticoid-free immunosuppressive regimen. New Engl J Med 343(4):230–238. 10.1056/nejm200007273430401 10911004 10.1056/NEJM200007273430401

[57] Brissova M, Niland JC, Cravens J, Olack B, Sowinski J, Evans-Molina C (2019) The Integrated Islet Distribution Program answers the call for improved human islet phenotyping and reporting of human islet characteristics in research articles. Diabetologia 62(7):1312–1314. 10.1007/s00125-019-4876-3 31089753 10.1007/s00125-019-4876-3 PMC7365209

[58] Qian D, Kaddis J, Niland JC (2007) A matching algorithm for the distribution of human pancreatic islets. Comput Stat Data Anal 51(12):5494–5506. 10.1016/j.csda.2007.02.030 22199413 10.1016/j.csda.2007.02.030 PMC3243971

[59] Niland JC, Stiller T, Cravens J, Sowinski J, Kaddis J, Qian D (2010) Effectiveness of a web-based automated cell distribution system. Cell Transplant 19(9):1133–1142. 10.3727/096368910x505486 20447343 10.3727/096368910X505486 PMC3242440

[60] Kaddis JS, Hanson MS, Cravens J et al (2013) Standardized transportation of human islets: an islet cell resource center study of more than 2,000 shipments. Cell Transplant 22(7):1101–1111. 10.3727/096368912x653219 22889479 10.3727/096368912X653219 PMC3745279

[61] Olack BJ, Alexander M, Swanson CJ et al (2020) Optimal time to ship human islets post tissue culture to maximize islet recovery. Cell Transplant 29:0963689720974582. 10.1177/0963689720974582 33231091 10.1177/0963689720974582 PMC7885128

[62] Kaddis JS, Danobeitia JS, Niland JC, Stiller T, Fernandez LA (2010) Multicenter analysis of novel and established variables associated with successful human islet isolation outcomes. Am J Transplant 10(3):646–656. 10.1111/j.1600-6143.2009.02962.x 20055802 10.1111/j.1600-6143.2009.02962.x PMC2860018

[63] Kayton NS, Poffenberger G, Henske J et al (2015) Human islet preparations distributed for research exhibit a variety of insulin-secretory profiles. Am J Physiol-Endocrinol Metab 308(7):E592-602. 10.1152/ajpendo.00437.2014 25648831 10.1152/ajpendo.00437.2014 PMC4385877

[64] Lohmann D, Jahr H, Verlohren H-J et al (1980) Insulin secretion in maturity-onset-diabetes function of isolated islets. Horm Metab Res 12(08):349–353. 10.1055/s-2007-996291 6997161 10.1055/s-2007-996291

[65] Verlohren H-J, Jahr H (1984) Insulin secretion in type II diabetics: in vivo and in vitro investigation. Exp Clin Endocrinol 83(02):216–224. 10.1055/s-0029-1210334 6373323 10.1055/s-0029-1210334

[66] Suleiman M, Sawatani T, Tesi M et al (2025) Functional recovery of islet β cells in human type 2 diabetes: transcriptome signatures unveil therapeutic approaches. Sci Adv 11(41):eads2905. 10.1126/sciadv.ads2905 41071888 10.1126/sciadv.ads2905 PMC12513425

[67] Meulebrouck S, Merrheim J, Queniat G et al (2024) Functional genetics reveals the contribution of delta opioid receptor to type 2 diabetes and beta-cell function. Nat Commun 15(1):6627. 10.1038/s41467-024-51004-6 39103322 10.1038/s41467-024-51004-6 PMC11300616

[68] Alonso L, Piron A, Morán I et al (2021) TIGER: the gene expression regulatory variation landscape of human pancreatic islets. Cell Rep 37(2):109807. 10.1016/j.celrep.2021.109807 34644572 10.1016/j.celrep.2021.109807 PMC8864863

[69] Gloyn AL, Ibberson M, Marchetti P et al (2022) Every islet matters: improving the impact of human islet research. Nat Metab 4(8):970–977. 10.1038/s42255-022-00607-8 35953581 10.1038/s42255-022-00607-8 PMC11135339

[70] Colli ML, Ramos-Rodríguez M, Nakayasu ES et al (2020) An integrated multi-omics approach identifies the landscape of interferon-α-mediated responses of human pancreatic beta cells. Nat Commun 11(1):2584. 10.1038/s41467-020-16327-0 32444635 10.1038/s41467-020-16327-0 PMC7244579

[71] Lablanche S, Borot S, Wojtusciszyn A et al (2021) Ten-year outcomes of islet transplantation in patients with type 1 diabetes: data from the Swiss-French GRAGIL network. Am J Transplant 21(11):3725–3733. 10.1111/ajt.16637 33961335 10.1111/ajt.16637

[72] Asplund O, Storm P, Chandra V et al (2022) Islet Gene View—a tool to facilitate islet research. Life Sci Alliance 5(12):e202201376. 10.26508/lsa.202201376 35948367 10.26508/lsa.202201376 PMC9366203

[73] Lopez-Noriega L, Callingham R, Martinez-Sánchez A et al (2025) Roles for the long non-coding RNA Pax6os1/PAX6-AS1 in pancreatic beta cell function. iScience 28(1):111518. 10.1016/j.isci.2024.111518 39811653 10.1016/j.isci.2024.111518 PMC11731260

[74] Mereu E, Balboa D, Liebig J et al (2025) Latent plasticity of the human pancreas across development, health, and disease. bioRxiv (Preprint). 3 Oct 2025. Available from: 10.1101/2025.10.01.679230

[75] Buemi A, Mourad NI, Ambroise J et al (2024) Donor- and isolation-related predictive factors of in vitro secretory function of cultured human islets. Front Endocrinol 15:1345351. 10.3389/fendo.2024.1345351 10.3389/fendo.2024.1345351 PMC10913008 38444584

[76] Wigger L, Barovic M, Brunner A-D et al (2021) Multi-omics profiling of living human pancreatic islet donors reveals heterogeneous beta cell trajectories towards type 2 diabetes. Nat Metab 3(7):1–15. 10.1038/s42255-021-00420-9 34183850 10.1038/s42255-021-00420-9

[77] Khamis A, Canouil M, Siddiq A et al (2019) Laser capture microdissection of human pancreatic islets reveals novel eQTLs associated with type 2 diabetes. Mol Metab 24:98–107. 10.1016/j.molmet.2019.03.004 30956117 10.1016/j.molmet.2019.03.004 PMC6531807

[78] Mezza T, Ferraro PM, Giuseppe GD et al (2021) Duodenopancreatectomy as a model to demonstrate the fundamental role of dysfunctional β cell in predicting diabetes. J Clin Investig 131(12):e146788. 10.1172/jci146788 33905373 10.1172/JCI146788 PMC8203447

[79] Cohrs CM, Panzer JK, Drotar DM et al (2020) Dysfunction of persisting β cells is a key feature of early type 2 diabetes pathogenesis. Cell Rep 31(1):107469. 10.1016/j.celrep.2020.03.033 32268101 10.1016/j.celrep.2020.03.033

[80] Marselli L, Sgroi DC, Bonner-Weir S, Weir GC (2009) Type 2 diabetes, methods and protocols. Methods Mol Biol 560:87–98. 10.1007/978-1-59745-448-3_8 19504246 10.1007/978-1-59745-448-3_8

[81] Gerst F, Jaghutriz BA, Staiger H et al (2018) The expression of aldolase B in islets is negatively associated with insulin secretion in humans. J Clin Endocrinol Metab 103(12):4373–4383. 10.1210/jc.2018-00791 30202879 10.1210/jc.2018-00791 PMC6915830

[82] Rajotte RV, Warnock GL, Evans MG, Ellis D, Dawidson I (1987) Isolation of viable islets of Langerhans from collagenase-perfused canine and human pancreata. Transplant Proc 19(1 Pt 2):918–922 3029913

[83] Warnock GL, Rajotte RV, Evans MG, Ellis D, DeGroot T, Dawidson I (1987) Isolation of islets of Langerhans following cold storage of human pancreas. Transplant Proc 19(4):3466–3468 3113011

[84] Warnock GL, Ellis DK, Cattral M, Untch D, Kneteman NM, Rajotte RV (1989) Viable purified islets of Langerhans from collagenase-perfused human pancreas. Diabetes 38(Suppl_1):136–139. 10.2337/diab.38.1.s136 10.2337/diab.38.1.s136 2535989

[85] Warnock GL, Kneteman NM, Ryan EA et al (1989) Continued function of pancreatic islets after transplantation in type I diabetes. Lancet 334(8662):570–572. 10.1016/s0140-6736(89)90701-0 10.1016/s0140-6736(89)90701-0 2570276

[86] Warnock GL, Kneteman NM, Rajotte RV (1990) Effect of diabetes on the function of transplanted human islets of Langerhans. Transplant Proc 22(2):804–805 2109405

[87] Warnock GL, Kneteman NM, Ryan E, Seelis REA, Rabinovitch A, Rajotte RV (1991) Normoglycaemia after transplantation of freshly isolated and cryopreserved pancreatic islets in type 1 (insulin-dependent) diabetes mellitus. Diabetologia 34(1):55–58. 10.1007/bf00404026 2055341 10.1007/BF00404026

[88] Warnock GI, Tsapogas P, Ryan EA et al (1995) Natural history of insulin independence after transplantation of multidonor cryopreserved pancreatic islets in type 1 diabetic humans. Transplant Proc 27(6):3159–3160 8539888

[89] Marfil-Garza BA, Hefler J, Verhoeff K et al (2023) Pancreas and islet transplantation: comparative outcome analysis of a single-centre cohort over 20-years. Ann Surg 277(4):672–680. 10.1097/sla.0000000000005783 36538619 10.1097/SLA.0000000000005783

[90] Kin T, O’Gorman D, Schroeder A et al (2011) Human islet distribution program for basic research at a single center. Transplant Proc 43(9):3195–3197. 10.1016/j.transproceed.2011.10.003 22099755 10.1016/j.transproceed.2011.10.003

[91] Kin T, O’Gorman D, Zhai W et al (2024) Contribution of a single islet transplant program to basic researchers in north America, Europe, and Asia through distributing human islets. OBM Transplant 08(02):1–15. 10.21926/obm.transplant.2402212

[92] Lyon J, Fox JEM, Spigelman AF et al (2016) Research-focused isolation of human islets from donors with and without diabetes at the alberta diabetes institute IsletCore. Endocrinology 157(2):560–569. 10.1210/en.2015-1562 26653569 10.1210/en.2015-1562

[93] Kolic J, Sun WG, Cen HH et al (2024) Proteomic predictors of individualized nutrient-specific insulin secretion in health and disease. Cell Metab 36(7):1619-1633.e5. 10.1016/j.cmet.2024.06.001 38959864 10.1016/j.cmet.2024.06.001 PMC11250105

[94] Barrett T, Wilhite SE, Ledoux P et al (2012) NCBI GEO: archive for functional genomics data sets—update. Nucleic Acids Res 41(D1):D991–D995. 10.1093/nar/gks1193 23193258 10.1093/nar/gks1193 PMC3531084

[95] D’Altri T, Freeberg MA, Curwin AJ et al (2025) The Federated European Genome-Phenome Archive as a global network for sharing human genomics data. Nat Genet 57(3):481–485. 10.1038/s41588-025-02101-9 40033058 10.1038/s41588-025-02101-9

[96] Deutsch EW, Bandeira N, Perez-Riverol Y et al (2022) The ProteomeXchange consortium at 10 years: 2023 update. Nucleic Acids Res 51(D1):D1539–D1548. 10.1093/nar/gkac1040 10.1093/nar/gkac1040 PMC9825490 36370099

[97] Mazzarelli JM, Brestelli J, Gorski RK et al (2007) EPConDB: a web resource for gene expression related to pancreatic development, beta-cell function and diabetes. Nucleic Acids Res 35(suppl_1):D751–D755. 10.1093/nar/gkl748 17071715 10.1093/nar/gkl748 PMC1781120

[98] Burren OS, Adlem EC, Achuthan P, Christensen M, Coulson RMR, Todd JA (2011) T1DBase: update 2011, organization and presentation of large-scale data sets for type 1 diabetes research. Nucleic Acids Res 39(suppl_1):D997–D1001. 10.1093/nar/gkq912 20937630 10.1093/nar/gkq912 PMC3013780

[99] Quayum N, Kutchma A, Sarkar SA et al (2008) GeneSpeed Beta Cell: an online genomics data repository and analysis resource tailored for the islet cell biologist. J Diabetes Res 2008(1):312060. 10.1155/2008/312060 10.1155/2008/312060 PMC2532782 18795106

[100] Costanzo MC, von Grotthuss M, Massung J et al (2023) The Type 2 Diabetes Knowledge Portal an open access genetic resource dedicated to type 2 diabetes and related traits. Cell Metab 35(4):695-710.e6. 10.1016/j.cmet.2023.03.001 10.1016/j.cmet.2023.03.001 PMC10231654 36963395

[101] Whetzel PL, Grethe JS, Banks DE, Martone ME (2015) The NIDDK information network: a community portal for finding data, materials, and tools for researchers studying diabetes, digestive, and kidney diseases. PLoS ONE 10(9):e0136206. 10.1371/journal.pone.0136206 26393351 10.1371/journal.pone.0136206 PMC4578941

[102] Mularoni L, Ramos-Rodríguez M, Pasquali L (2017) The pancreatic islet regulome browser. Front Genet 8:13. 10.3389/fgene.2017.00013 28261261 10.3389/fgene.2017.00013 PMC5306130

[103] Saunders DC, Messmer J, Kusmartseva I et al (2020) Pancreatlas: applying an adaptable framework to map the human pancreas in health and disease. Patterns 1(8):100120. 10.1016/j.patter.2020.100120 33294866 10.1016/j.patter.2020.100120 PMC7691395

[104] Hart NJ, Aramandla R, Poffenberger G et al (2018) Cystic fibrosis–related diabetes is caused by islet loss and inflammation. Jci Insight 3(8):e98240. 10.1172/jci.insight.98240 29669939 10.1172/jci.insight.98240 PMC5931120

[105] Driver JP, Chen Y-G, Mathews CE (2012) Comparative genetics: synergizing human and NOD mouse studies for identifying genetic causation of type 1 diabetes. Rev Diabet Stud 9(4):169–187. 10.1900/rds.2012.9.169 23804259 10.1900/RDS.2012.9.169 PMC3740689

[106] Segerstolpe Å, Palasantza A, Eliasson P et al (2016) Single-cell transcriptome profiling of human pancreatic islets in health and type 2 diabetes. Cell Metab 24(4):593–607. 10.1016/j.cmet.2016.08.020 27667667 10.1016/j.cmet.2016.08.020 PMC5069352

[107] Ortega HI, Udler MS, Gloyn AL, Sharp SA (2025) Diabetes mellitus polygenic risk scores: heterogeneity and clinical translation. Nat Rev Endocrinol 21(9):530–545. 10.1038/s41574-025-01132-w 40467969 10.1038/s41574-025-01132-w PMC12614124

[108] Kaestner KH, Powers AC, Naji A, Consortium H, Atkinson MA (2019) NIH initiative to improve understanding of the pancreas, islet, and autoimmunity in type 1 diabetes: the Human Pancreas Analysis Program (HPAP). Diabetes 68(7):1394–1402. 10.2337/db19-0058 31127054 10.2337/db19-0058 PMC6609987

[109] Shapira SN, Naji A, Atkinson MA, Powers AC, Kaestner KH (2022) Understanding islet dysfunction in type 2 diabetes through multidimensional pancreatic phenotyping: The Human Pancreas Analysis Program. Cell Metab 34(12):1906–1913. 10.1016/j.cmet.2022.09.013 36206763 10.1016/j.cmet.2022.09.013 PMC9742126

[110] Benhayon H, Yi X, Schyr RB, Eizirik DL, Ben-Zvi D (2025) A web tool for easy and versatile analysis of human endocrine pancreas single-cell RNAseq data. Diabetes Obes Metab 28(2):1545–1549. 10.1111/dom.70281 10.1111/dom.70281 PMC12803539 41208596

[111] Ewald JD, Lu Y, Ellis CE et al (2025) HumanIslets.com: improving accessibility, integration, and usability of human research islet data. Cell Metab 37(1):7–11. 10.1016/j.cmet.2024.09.001 39357523 10.1016/j.cmet.2024.09.001 PMC12577030

[112] dos Santos T, Dai X-Q, Jones RC et al (2025) Altered immune and metabolic molecular pathways drive islet cell dysfunction in human type 1 diabetes. J Clin Invest 135(23):e195267. 10.1172/jci195267 10.1172/JCI195267 PMC12646676 41026524

[113] Dai X-Q, Camunas-Soler J, Briant LJB et al (2022) Heterogenous impairment of α cell function in type 2 diabetes is linked to cell maturation state. Cell Metab 34(2):256-268.e5. 10.1016/j.cmet.2021.12.021 35108513 10.1016/j.cmet.2021.12.021 PMC8852281

[114] Piron A, Szymczak F, Folon L et al (2025) Identification of novel type 1 and type 2 diabetes genes by co-localization of human islet eQTL and GWAS variants with colocRedRibbon. Cell Genom 5(11):101004. 10.1016/j.xgen.2025.101004 40961947 10.1016/j.xgen.2025.101004 PMC12648100

[115] Mizukami H, Takahashi K, Inaba W et al (2014) Age-associated changes of islet endocrine cells and the effects of body mass index in Japanese. J Diabetes Invest 5(1):38–47. 10.1111/jdi.12118 10.1111/jdi.12118 PMC4025233 24843735

[116] Toledo MP, Xie G, Wang YJ (2024) Comprehensive characterization of islet remodeling in development and in diabetes using mass cytometry. Endocrinology 165(9):bqae094. 10.1210/endocr/bqae094 39058908 10.1210/endocr/bqae094

[117] Henquin J (2018) Influence of organ donor attributes and preparation characteristics on the dynamics of insulin secretion in isolated human islets. Physiol Rep 6(5):e13646. 10.14814/phy2.13646 29536672 10.14814/phy2.13646 PMC5849575

[118] D’Aleo V, Guerra SD, Gualtierotti G et al (2010) Functional and survival analysis of isolated human islets. Transplant Proc 42(6):2250–2251. 10.1016/j.transproceed.2010.05.132 20692456 10.1016/j.transproceed.2010.05.132

[119] Evans-Molina C, Pettway YD, Saunders DC et al (2025) Heterogeneous endocrine cell composition defines human islet functional phenotypes. bioRxiv (Preprint). 25 Aug 2025. Available from: 10.1101/2024.11.20.623809 10.1038/s41467-026-70689-5 PMC13168233 42120367

[120] Hart NJ, Powers AC (2019) Use of human islets to understand islet biology and diabetes: progress, challenges and suggestions. Diabetologia 62(2):212–222. 10.1007/s00125-018-4772-2 30547228 10.1007/s00125-018-4772-2 PMC6325002

[121] Poitout V, Satin LS, Kahn SE et al (2019) A call for improved reporting of human islet characteristics in research articles. Diabetologia 62(2):209–211. 10.1007/s00125-018-4784-y 30547229 10.1007/s00125-018-4784-y PMC7259467

[122] Lyon JG, Carr AL, Smith NP et al (2024) Human research islet cell culture outcomes at the Alberta Diabetes Institute IsletCore. Islets 16(1):2385510. 10.1080/19382014.2024.2385510 39097865 10.1080/19382014.2024.2385510 PMC11299626

[123] Wang S, Flibotte S, Camunas-Soler J, MacDonald PE, Johnson JD (2021) A new hypothesis for type 1 diabetes risk: the at-risk allele at rs3842753 associates with increased beta-cell INS messenger RNA in a meta-analysis of single-cell RNA-sequencing data. Can J Diabetes 45(8):775-784.e2. 10.1016/j.jcjd.2021.03.007 34052132 10.1016/j.jcjd.2021.03.007

[124] Yong HJ, Toledo MP, Nowakowski RS, Wang YJ (2022) Sex differences in the molecular programs of pancreatic cells contribute to the differential risks of type 2 diabetes. Endocrinology 163(11):bqac156. 10.1210/endocr/bqac156 36130190 10.1210/endocr/bqac156 PMC10409906

[125] Song Y, He C, Jiang Y et al (2023) Bulk and single-cell transcriptome analyses of islet tissue unravel gene signatures associated with pyroptosis and immune infiltration in type 2 diabetes. Front Endocrinol 14:1132194. 10.3389/fendo.2023.1132194 10.3389/fendo.2023.1132194 PMC10034023 36967805

[126] Viñuela A, Varshney A, van de Bunt M et al (2020) Genetic variant effects on gene expression in human pancreatic islets and their implications for T2D. Nat Commun 11(1):4912. 10.1038/s41467-020-18581-8 32999275 10.1038/s41467-020-18581-8 PMC7528108

[127] Petrenko V, Gandasi NR, Sage D, Tengholm A, Barg S, Dibner C (2020) In pancreatic islets from type 2 diabetes patients, the dampened circadian oscillators lead to reduced insulin and glucagon exocytosis. Proc Natl Acad Sci 117(5):2484–2495. 10.1073/pnas.1916539117 31964806 10.1073/pnas.1916539117 PMC7007532

[128] Petrenko V, Sinturel F, Loizides-Mangold U et al (2022) Type 2 diabetes disrupts circadian orchestration of lipid metabolism and membrane fluidity in human pancreatic islets. PLoS Biol 20(8):e3001725. 10.1371/journal.pbio.3001725 35921354 10.1371/journal.pbio.3001725 PMC9348689

[129] Flanagin A, Frey T, Christiansen SL, AMA Manual of Style Committee (2021) Updated guidance on the reporting of race and ethnicity in medical and science journals. JAMA 326(7):621–627. 10.1001/jama.2021.13304 34402850 10.1001/jama.2021.13304

[130] Doliba NM, Roman J, Rozo AV et al (2022) Ethnic differences in pancreatic hormone secretion in health and T2D. Diabetes 71(Suppl 1):253-LB. 10.2337/db22-253-LB

[131] Katte JC, Squires S, Dehayem MY et al (2025) Non-autoimmune, insulin-deficient diabetes in children and young adults in Africa: evidence from the Young-Onset Diabetes in sub-Saharan Africa (YODA) cross-sectional study. Lancet Diabetes Endocrinol 13(9):745–753. 10.1016/s2213-8587(25)00120-2 40706606 10.1016/S2213-8587(25)00120-2

[132] Misra S (2025) Ethnic diversity in precision medicine: a reality or an aspiration? Diabetologia 68(11):2449–2464. 10.1007/s00125-025-06513-4 40773074 10.1007/s00125-025-06513-4 PMC12534348

[133] Gombault C, Grenet G, Segurel L et al (2023) Population designations in biomedical research: limitations and perspectives. HLA 101(1):3–15. 10.1111/tan.14852 36258305 10.1111/tan.14852 PMC10099491

[134] Wright AK, Welsh P, Gill JMR et al (2020) Age-, sex- and ethnicity-related differences in body weight, blood pressure, HbA1c and lipid levels at the diagnosis of type 2 diabetes relative to people without diabetes. Diabetologia 63(8):1542–1553. 10.1007/s00125-020-05169-6 32435821 10.1007/s00125-020-05169-6 PMC7351865

[135] Luk AOY, Fan Y, Fan B, Chow EWK, O TCK (2025) Heterogeneity in the development of diabetes-related complications: narrative review of the roles of ancestry and geographical determinants. Diabetologia 68(11):2386–2404. 10.1007/s00125-025-06482-8 40696182 10.1007/s00125-025-06482-8 PMC12534336

[136] Rogic S, Yu XX, Xu B et al (2025) Persistent hindrances to data re-use in single-cell genomics. bioRxiv (Preprint). 3 Oct 2025. Available from: 10.1101/2025.10.02.680150

[137] Mawla AM, Huising MO (2019) Navigating the depths and avoiding the shallows of pancreatic islet cell transcriptomes. Diabetes 68(7):1380–1393. 10.2337/dbi18-0019 31221802 10.2337/dbi18-0019 PMC6609986

[138] Bosi E, Marselli L, Luca CD et al (2020) Integration of single-cell datasets reveals novel transcriptomic signatures of β-cells in human type 2 diabetes. NAR Genom Bioinform 2(4):lqaa097. 10.1093/nargab/lqaa097 33575641 10.1093/nargab/lqaa097 PMC7679065

[139] Molnár-Gábor F, Korbel JO (2020) Genomic data sharing in Europe is stumbling—could a code of conduct prevent its fall? EMBO Mol Med 12(3):e11421. 10.15252/emmm.201911421 32072760 10.15252/emmm.201911421 PMC7059003

[140] Zhou G, Pang Z, Lu Y, Ewald J, Xia J (2022) OmicsNet 2.0: a web-based platform for multi-omics integration and network visual analytics. Nucleic Acids Res 50(W1):W527–W533. 10.1093/nar/gkac376 35639733 10.1093/nar/gkac376 PMC9252810

[141] Ewald JD, Zhou G, Lu Y et al (2024) Web-based multi-omics integration using the Analyst software suite. Nat Protoc 19(5):1–31. 10.1038/s41596-023-00950-4 38355833 10.1038/s41596-023-00950-4

[142] Kruse ARS, Lardenoije R, Migas LG et al (2025) Integrative spatial omics for systems-level mapping of pathological niches. bioRxiv (Preprint). 18 Oct 2025. Available from: 10.1101/2025.09.12.675904

