# Supplementary material for: From pancreas and islet resources to diabetes insights
Source: Diabetologia. 2026 May 1;69(7):1741–58. doi: 10.1007/s00125-026-06731-4 (PMC13236752; doi:10.1007/s00125-026-06731-4)
Supplement: Supplementary file 1 — Slideset of figures (PPTX 659 KB) [file 125_2026_6731_MOESM1_ESM.pptx]

## Slide 1
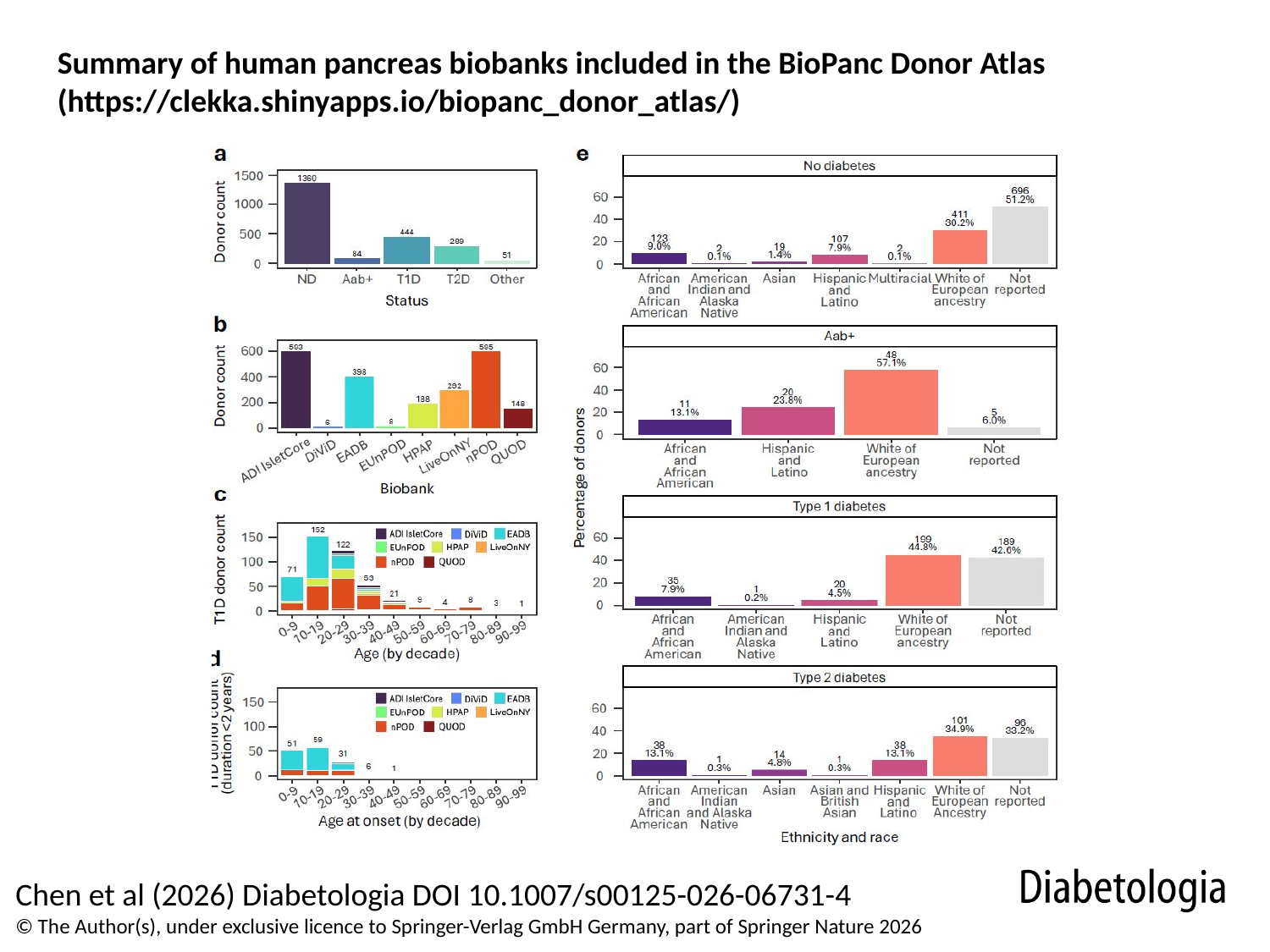

Summary of human pancreas biobanks included in the BioPanc Donor Atlas (https://clekka.shinyapps.io/biopanc_donor_atlas/)
Chen et al (2026) Diabetologia DOI 10.1007/s00125-026-06731-4
© The Author(s), under exclusive licence to Springer-Verlag GmbH Germany, part of Springer Nature 2026

## Slide 2
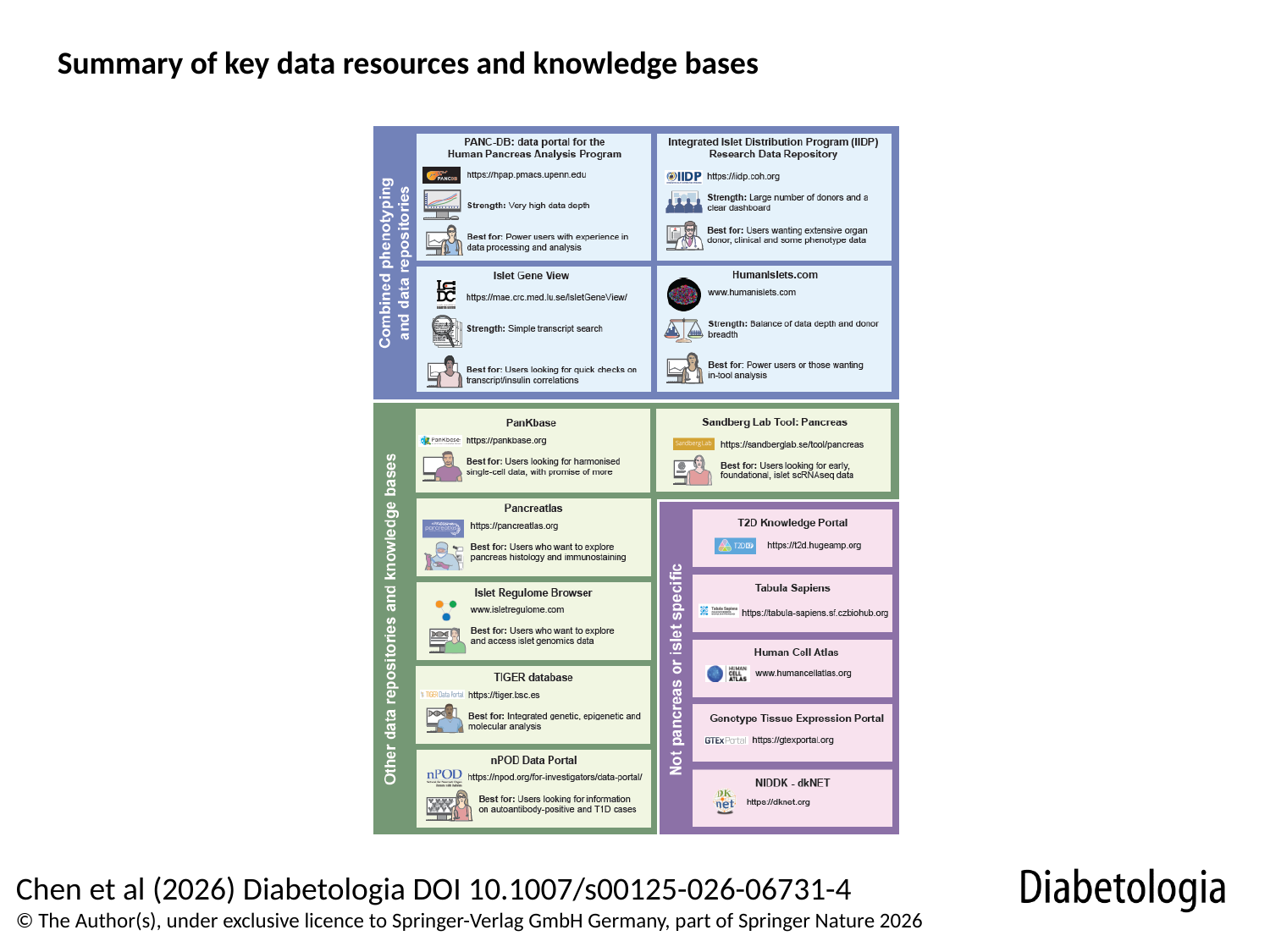

Summary of key data resources and knowledge bases
Chen et al (2026) Diabetologia DOI 10.1007/s00125-026-06731-4
© The Author(s), under exclusive licence to Springer-Verlag GmbH Germany, part of Springer Nature 2026

## Slide 3
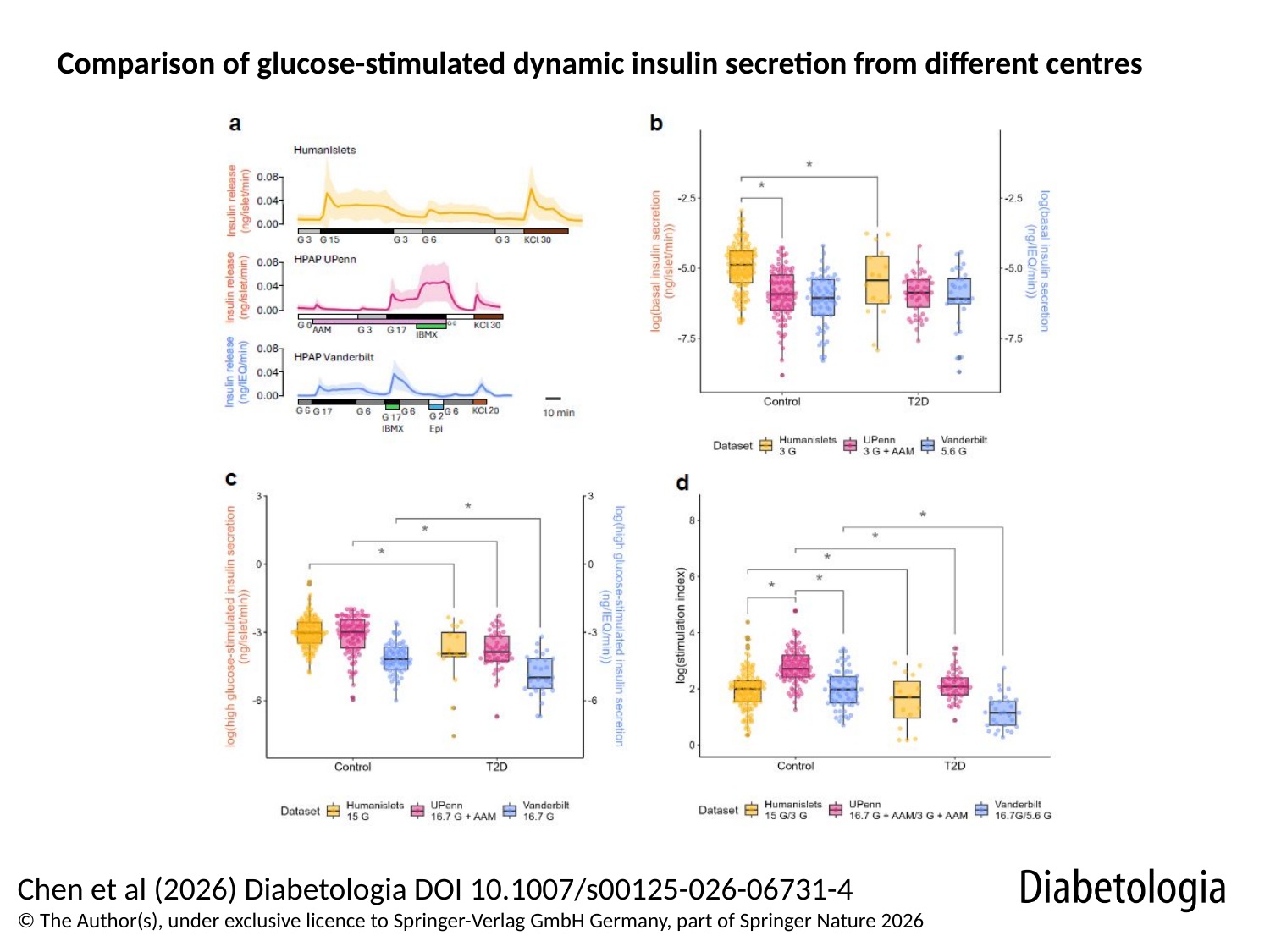

Comparison of glucose-stimulated dynamic insulin secretion from different centres
Chen et al (2026) Diabetologia DOI 10.1007/s00125-026-06731-4
© The Author(s), under exclusive licence to Springer-Verlag GmbH Germany, part of Springer Nature 2026
